# New Insights Into Mitochondrial Dysfunction at Disease Susceptibility Loci in the Development of Type 2 Diabetes

**DOI:** 10.3389/fendo.2021.694893

**Published:** 2021-08-11

**Authors:** Hannah Maude, Winston Lau, Nikolas Maniatis, Toby Andrew

**Affiliations:** ^1^Department of Metabolism, Digestion & Reproduction, Imperial College, London, United Kingdom; ^2^Department of Genetics, Evolution and Environment, University College London, London, United Kingdom

**Keywords:** mitochondrial dysfunction, type 2 diabetes, insulin action and resistance, adipose tissue, gene set enrichment analyses, differential gene expression, heritable susceptibility

## Abstract

This study investigated the potential genetic mechanisms which underlie adipose tissue mitochondrial dysfunction in Type 2 diabetes (T2D), by systematically identifying nuclear-encoded mitochondrial genes (NEMGs) among the genes regulated by T2D-associated genetic loci. The target genes of these ‘disease loci’ were identified by mapping genetic loci associated with both disease and gene expression levels (expression quantitative trait loci, eQTL) using high resolution genetic maps, with independent estimates co-locating to within a small genetic distance. These co-locating signals were defined as T2D-eQTL and the target genes as T2D *cis-*genes. In total, 763 *cis-*genes were associated with T2D-eQTL, of which 50 were NEMGs. Independent gene expression datasets for T2D and insulin resistant cases and controls confirmed that the *cis-*genes and *cis-*NEMGs were enriched for differential expression in cases, providing independent validation that genetic maps can identify informative functional genes. Two additional results were consistent with a potential role of T2D-eQTL in regulating the 50 identified *cis-*NEMGs in the context of T2D risk: (1) the 50 *cis-*NEMGs showed greater differential expression compared to other NEMGs and (2) other NEMGs showed a trend towards significantly decreased expression if their expression levels correlated more highly with the subset of 50 *cis-*NEMGs. These 50 *cis*-NEMGs, which are differentially expressed and associated with mapped T2D disease loci, encode proteins acting within key mitochondrial pathways, including some of current therapeutic interest such as the metabolism of branched-chain amino acids, GABA and biotin.

## Introduction

**Figure 1:**
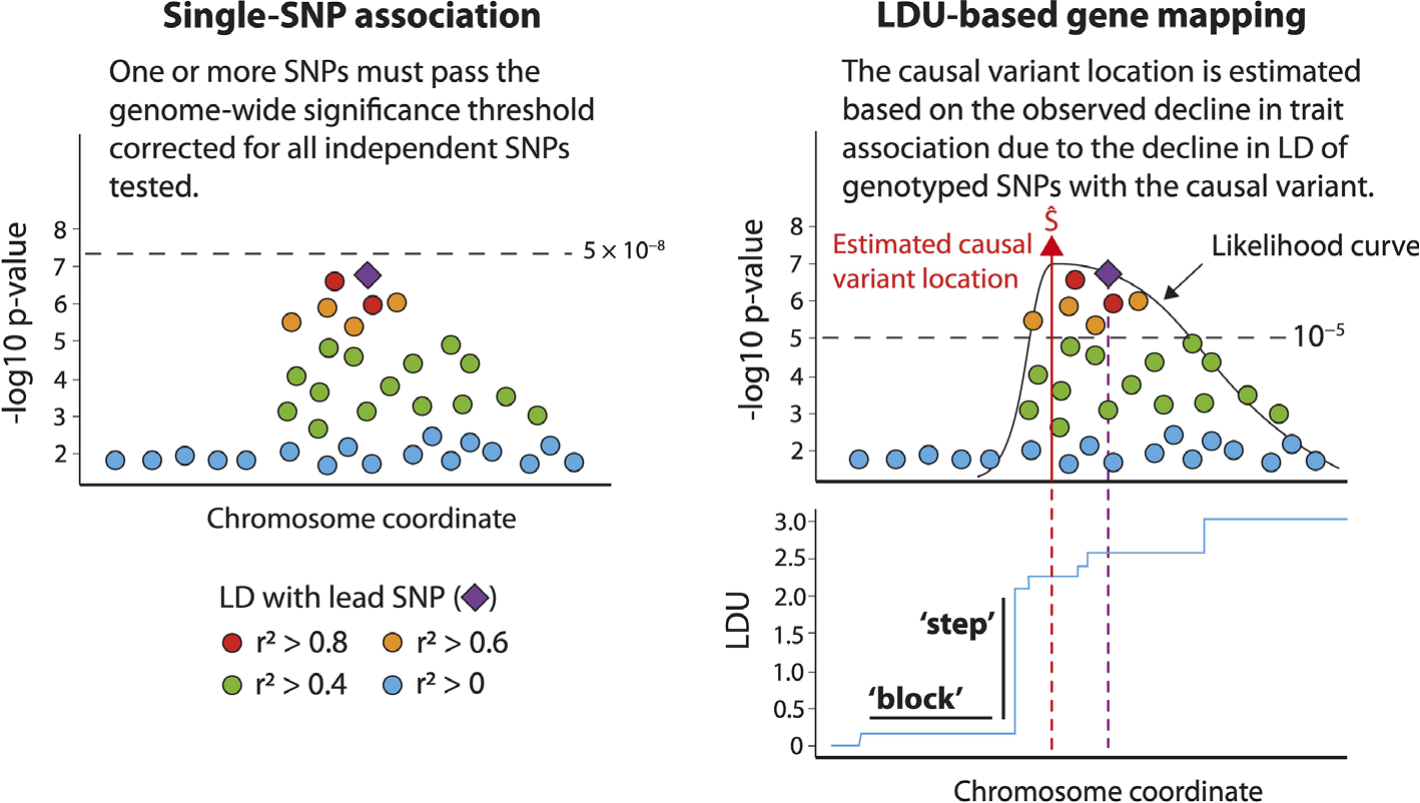
An overview of linkage disequilibrium unit (LDU)-based gene mapping and single-SNP tests of association. Single-SNP methods test each SNP independently for association and detect disease loci where one or more SNPs achieve a genome-wide significance threshold of 5 × 10^-8^, typically using a regression that tests for differences in case *vs* control allele frequencies. LDU-based gene mapping detects disease loci where multiple genetic markers display a pattern of association which reflects the decline in LD between SNPs and the estimated location of a causal variant. LD information is obtained from a relevant population-specific genetic LDU map shown in blue, where horizontal ‘blocks’ represent extended LD and vertical ‘steps’ represent a breakdown in LD. Note that depending upon the local LD structure, the lead SNP and causal variant estimated location are rarely the same.

Considerable efforts have been spent in a bid to understand the genetic mechanisms that underpin Type 2 diabetes (T2D) and other complex diseases. Dozens of genome wide association studies (GWAS) have successfully replicated the association of hundreds of genetic markers with T2D (referred to as disease loci) using single-SNP (single-nucleotide polymorphism) tests for association (1), with recent studies achieving cohort sizes of up to one million participants (2). Such large cohorts enable variants with small phenotypic effects to achieve statistical significance, although introduce additional problems of potential heterogeneity and ability to obtain high quality phenotype data. We previously used an alternative test for association, here called LDU-based gene mapping, to map 111 novel T2D disease loci at genome-wide significance in a cohort of 5,800 T2D cases and 9,691 controls (3). 20 of these loci were more recently also reported in a GWAS using single-SNP tests of association (based on a replication co-location criterion of 500kb), but required over one million participants to achieve statistical significance (2). LDU-based gene mapping, described in detail elsewhere (4), captures additional information at each locus by utilizing high-resolution genetic maps measured in additive linkage disequilibrium units (LDU) (3) (**Figure 1**), is robust to imputation errors by using only observed genotyped marker panels, and requires a lower statistical threshold by correcting for ~5,000 genomic tests. In this current study we show that the LDU-based method can be applied to investigate a specific biological hypothesis in the context of disease, namely the role of mitochondrial function in T2D.

Mitochondrial function in T2D is of therapeutic interest since the mitochondria are targeted by multiple drugs used to treat T2D (5). Mitochondrial dysfunction, for which definitions include differences in numbers, morphology, gene expression, oxidative phosphorylation, substrate oxidation and production of reactive oxygen species (ROS) or ATP, has been well established to be implicated in T2D through observational and functional studies [see several detailed reviews (6–9)]. This is unsurprising, since insulin activity and therefore resistance can regulate mitochondrial function (10, 11). On the other hand, manipulating mitochondrial function both *in vitro* and *in vivo* can influence insulin sensitivity (12–15), while mutations in the mitochondrial genome cause diabetes syndromes in humans (16–19). Thus, the question remains to what extent, if any, the mitochondrial dysfunction observed in individuals with T2D and their healthy offspring (20–24) may causally contribute to disease (25). Since inherited genetic mechanisms can precede and increase risk of disease onset, we used a novel genetic design to identify potential mechanisms which drive mitochondrial dysfunction in the context of T2D. Specifically, this study investigated T2D disease loci which regulate the expression levels of nuclear-encoded mitochondrial genes (NEMGs) in adipose tissue, since mitochondrial dysfunction in adipose can result in ectopic fat production, inflammation (26) and peripheral insulin resistance (IR) (27, 28). NEMGs, through which T2D genetic risk variants may feasibly regulate mitochondrial function, are particularly enriched for regulation by genetic variants in adipose tissue (29).

Here, we aim to identify NEMGs among the target genes (*cis-*genes) regulated by T2D disease loci. Since T2D loci are overwhelmingly non-coding (1, 30, 31), *cis-*genes were here defined based on the colocation of disease loci with adipose tissue eQTL (expression quantitative trait loci), independently mapped using LDU-based gene mapping, under the hypothesis of shared functional variants. This current study follows our previous observation that the *cis*-genes of 111 novel T2D disease loci (hereafter referred to as T2D *cis-*genes) included NEMGs (3). Here, the list of T2D *cis-*genes was extended and refined based on the co-location of eQTL within a small genetic distance of disease loci, to effectively exclude eQTL which appeared physically close (separated by a small base pair distance) but represented independent association signals from variants separated by LD breakdown (and therefore a large genetic distance). The extended list of T2D *cis*-genes was used to address two fundamental research objectives: (1) to validate the identified T2D *cis*-genes and *cis*-NEMGs, by directly testing independent gene expression datasets for evidence that these genes associate with T2D and insulin resistance, by means of differential expression compared to healthy controls and (2) to identify mitochondrial pathways potentially dysregulated in the context of T2D.

## Materials and Methods

### T2D and eQTL Location Estimates

The current study analyzed location estimates of causal variants associated with T2D and adipose gene expression, mapped as previously described by Lau et al. (3) using an LDU-based multi-marker test of association described in detail elsewhere (4) (see **Figure 1** for a brief summary). The multi-marker test of association implements an adapted Malécot model to estimate causal variant locations within 4,800 distinct analytical windows (each approximately 10 LDU in size) comprising the autosomal genome. One test is fitted per window and incorporates all genotyped variants, testing for declines in SNP-trait association as would be expected for SNPs in decreasing LD with a causal variant. Decline in LD is modelled based upon population-specific, high-resolution genetic maps measured in metric additive LDU and constructed using millions of observed genotyped markers for population-specific reference panels (3, 32). Compared to single-SNP tests, which test every SNP independently, LDU-based mapping effectively reduces the total number of association tests and genome-wide significance threshold (see **Figure 1**), while controlling for Type I and Type II error rates (4).

The locations of causal variants associated with T2D (*Ŝ_T2D_*) were estimated from two European case-control cohorts (the Wellcome Trust Case Control Consortium, WTCCC1 and WTCCC2) (33, 34) and one African American (AA) cohort (obtained from the National Institute of Diabetes and Digestive and Kidney Diseases, NIDDK) (35), totaling 5,800 cases and 9,691 controls. Genetic locations associated with adipose tissue gene expression levels (*Ŝ_eQTL_*) were also mapped for genes within ±1.5Mb of replicated *Ŝ_T2D_*, defined as co-locating within 1LDU, using a population-based sample of healthy Europeans from the MuTHER Consortium (36). The current study included *Ŝ_T2D_* using a nominal significance threshold (*Ŝ_T2D_* α < 1x10^-3^) in order to expand the previously reported list of *cis*-genes (3) defined using Bonferroni-correction (*Ŝ_T2D_* α < 1x10^-5^, corrected for ~5,000 independent tests) and facilitate pathway enrichment analysis. *Ŝ_eQTL_* were included if they had a nominal p-value <0.05.

**Figure 2:**
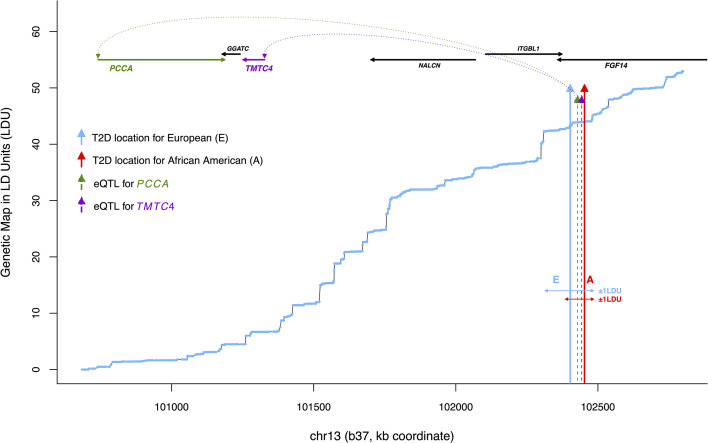
An example of co-locating T2D and eQTL locations, or ‘T2D-eQTL’ (chr13). Plotted is physical distance (kb coordinate) against genetic distance in linkage disequilibrium units (LDU), based on a European genetic map constructed by Lau et al. (3). The blue line shows cumulative LDU across the region in a ‘step’ and ‘block’ structure, corresponding to regions of LD breakdown and extended LD, respectively. Blue and red arrows correspond to *Ŝ_T2D_* location estimates associated with T2D in the Wellcome Trust European and African American populations (WTC and AA), respectively, with horizontal arrows illustrating the asymmetrical physical distance extending ±1 LDU. The dotted arrows show *Ŝ_eQTL_* location estimates for *two cis*-genes, *PCCA* which is a nuclear-encoded mitochondrial gene (NEMG) and *TMTC4*, which is not (confidence intervals not shown). All *Ŝ_T2D_* and *Ŝ_eQTL_* location estimates are provided in **Table 1**.

*Ŝ_T2D_* and *Ŝ_eQTL_* were integrated in this study to identify putative T2D-eQTL based upon a co-location threshold of 1 LDU. This threshold reflects a small genetic distance and excludes genetically distinct signals (similar to replicating lead SNPs based on high LD). 1 LDU corresponds to a median distance of 48kb per chromosome, ranging from a median of 34kb (chr22) to 57kb (chr2), as defined by the European genetic LDU map. Replicated T2D loci were defined where two or more *Ŝ_T2D_* co-located within 1 LDU and were considered cosmopolitan if one *Ŝ_T2D_* was AA (converted to the European LDU map *via* its physical location). Adipose tissue *Ŝ_eQTL_* within 1 LDU of a replicated *Ŝ_T2D_*, referred to as ‘T2D-eQTL’ (an example is illustrated in **Figure 2**), were used to define target T2D *cis-*genes. Gene expression probes associated with *Ŝ_eQTL_* which overlapped multiple genes were assigned gene names using the annotated gene (according to the R package *illuminaHumanv3.db*). Otherwise, gene names were assigned using Ensembl GRCh37 gene coordinates. Ensembl gene identifiers were converted to HGNC identifiers using the *biomaRt* package in R (37). All LDU location estimates have been converted to physical location (GRCh37 or b37) for presentational purposes.

### Identification of *Cis*-Regulated Nuclear Encoded Mitochondrial Genes

NEMGs were identified by cross-referencing Ensembl and HGNC gene identifiers with the combined MitoCarta2.0 and MitoCarta+ databases (38, 39) of known NEMGs. KEGG pathways and KEGG ontology terms (40) were used to group the *cis*-NEMGs by function, in addition to GeneCards summaries where KEGG data were lacking (41). Additional databases including STRING (42), Refseq (43), Reactome (44) and Gene Ontology (45) were used to further discuss potential gene functions.

### Validation of T2D *Cis*-Genes: Case-Control Differential Gene Expression

Independent datasets with baseline gene expression data for T2D and IR case-control cohorts, measured using Affymetrix arrays, were identified from the Gene Expression Omnibus (GEO) database (46, 47). The search string is shown in the **Supplementary Methods**, with inclusion and exclusion criteria listed in **Supplementary Table S2**. **Supplementary Figure S1** shows the datasets excluded following full-text review. The tissues skeletal muscle, liver and pancreas were included in addition to subcutaneous adipose to investigate potential cross-tissue effects on *cis-*gene expression. Raw data were downloaded as CEL files and normalized using robust multi-array averaging (RMA) (48), as implemented by the R package *oligo* (49). Phenotype data was extracted using *GEOquery* (50) and the appropriate array annotation package. Gene-centric Z scores, used as a summary measure of differential expression for each gene by disease status, were calculated by regressing normalized probe expression on phenotype, coded 0 for control and 1 for T2D or IR cases. Linear mixed-effects regression models (including intercept random effects) were fitted to model probes for genes with more than one annotated probe. This model reflects a conservative assumption that the probes for each gene share the same direction of effect (where untrue, the effect will be expected to be reduced or go undetected). Where available, age and BMI were included as co-variates. The case-control datasets were meta-analyzed for each tissue using a random-effects model as described by Choi et al. (51) and implemented in the R package *geneMeta* (52). One dataset, accession GSE25462 (53), included skeletal muscle gene expression data for normoglycemic but IR individuals with one or two parents with T2D (n = 15 and n = 10, respectively), as well as healthy individuals with no family history (n = 10). Gene expression levels were regressed against the number of parents with T2D (0, 1 or 2), in order to separately investigate potential genetic risk without confounding due to disease onset.

### Differential Expression of T2D *Cis-*Genes: Gene Set Enrichment Analysis

Gene-set significance was calculated using a non-parametric Wilcoxon rank sum test, previously shown to have the highest reproducibility and sensitivity compared to other GSEA tests (54), and gene-level sampling with 10,000 permutations. False discovery rate (FDR) was used to correct for multiple testing. GSEA was applied to genome-wide Z scores using the R package *piano* (55).

### Differential Expression of ‘Non-T2D’ NEMGs and the Correlation of Gene Expression With the T2D *Cis-*NEMGs

Since the expression of NEMGs is highly correlated, there is likely to be limited power to detect an enrichment of differential expression for the subset of T2D *cis-*NEMGs compared to the background of all NEMGs (tested using GSEA as described above). The case *vs* control expression of NEMGs in either mitoCarta2.0 or mitoCarta+ and *not* identified as T2D *cis-*genes in this study (‘non-T2D’ NEMGs) was further investigated in relation to how highly their expression correlated with the subset of identified T2D *cis-*NEMGs. The null hypothesis is that there is no relationship between the degree of NEMG differential expression and their strength of correlation with the mapped T2D *cis-*NEMGs, while significant association would demonstrate that T2D-eQTL association distinguishes between NEMG differential expression in cases. Differential expression was measured as the gene expression summary Z-scores from the adipose tissue meta-analysis. All non-T2D NEMGs were correlated with the expression of each T2D *cis-*NEMG to calculate pairwise Pearson correlation coefficients and the mean value estimated for the T2D *cis-*NEMGs as a group. For this analysis, the gene expression correlation coefficients were measured using the control-only normalized expression values from the adipose dataset GSE27949, having the largest number of controls at n = 11, with gene-level expression calculated as the average of the annotated probes. For all non-T2D NEMGs, the mean correlation value was regressed against the adipose Z-score of differential expression using linear regression. Mean gene expression was included in the regression as a covariate.

### Characterization of T2D *Cis*-Genes: Enrichment of Mitochondrial Pathways by Gene Count

41 mitochondrial-related gene sets were identified and downloaded from the curated Molecular Signatures Database (MSigDB) (56, 57), each with >25% overlap with the combined MitoCarta2.0/MitoCarta+ databases of NEMGs (38, 39). The gene sets (listed in the **Supplementary Table S6**) were tested for enrichment in the total T2D *cis*-genes. For each gene set, the observed number of T2D *cis*-genes was compared to expected counts under the null. Expected counts were generated using two alternative permutation-approaches, random and structured. For the random approach, control genes (equal to the total number of identified T2D *cis-*genes) were randomly selected from the genomic background and for the structured approach, genes were randomly selected from within ±1.5Mb of each other to control for any potential local structure (an average of 4.6 *cis*-genes were observed per T2D-eQTL). Empirical p-values were based on 10,000 permutations. Gene sets which showed nominal evidence of differential expression (p-value < 0.05) were also tested for case-control differential gene expression using GSEA, as described above.

## Results

### Mapping T2D-eQTL and *Cis*-NEMGs

This study analyzed genomic location estimates of causal variants associated with T2D (*Ŝ_T2D_*) and adipose gene expression (*Ŝ_eQTL_*) mapped as previously described by Lau et al. (3) using LDU-based gene mapping (see Methods and **Figure 1**). *Ŝ_T2D_* estimates were mapped in two European and one African American T2D case-control cohorts and were required to replicate in at least two datasets. The list of previously mapped loci was here expanded to include nominally significant loci to facilitate pathway enrichment analysis of target genes. Crucially, *Ŝ_T2D_* replication was defined using a stringent co-location threshold, such that *Ŝ_T2D_* were independently mapped within a genetic distance of 1 LDU [according to a European-specific genetic LDU map assembled by Lau et al. (3)]. This approach aims to prioritize *Ŝ_T2D_* estimates which are likely to represent shared causal variants, while excluding *Ŝ_T2D_* which are physically close in chromosome (base pair) location but are separated by a large genetic distance and LD breakdown (and therefore capture independently inherited variants). **Figure 2**, which shows the genetic LDU map, illustrates how the kb (kilobase pair) distance corresponding to 1 LDU can differ depending on the genomic location, with 1 LDU extending across a larger kb distance where there is more extensive LD between pairs of genetic markers, for example due to a lack of recombination (32). Across the autosomal genome, 1 LDU corresponds to a median of 48kb per chromosome and ranges from a median of 34kb (chr22) to 57kb (chr2).

A total of 174 loci showed replicated association with T2D based on a 1 LDU threshold. To identify the likely target genes (*cis-*genes) regulated by these loci, adipose tissue eQTL were mapped using LDU-based gene mapping for all genes within 1.5Mb. A total of 763 genes were associated with nominally significant *Ŝ_eQTL_* estimates (likely location of causal variants associated with gene expression levels) within 1 LDU of 167 T2D loci, of which 59 were European-specific loci and 108 were shared with African Americans. Co-locating T2D loci and eQTL are referred to as ‘T2D-eQTL’ and associated genes as ‘T2D *cis-*genes’. **Figure 2** illustrates an example T2D-eQTL, where an eQTL associated with the neighboring gene *PCCA* co-located with two independent *Ŝ_T2D_*. The genetic LDU *vs* physical kb coordinates of the region are plotted to illustrate the 1 LDU distance used to define co-location and shared T2D-eQTL signals. Since the extent of 1 LDU depends on the pairwise LD between SNPs at the genomic locus, the 1 LDU distance in **Figure 2** can be seen to extend further downstream of the African American T2D location (red arrow) due to an upstream breakdown in LD at this locus.

Based on comparison with the MitoCarta2.0 (38) and MitoCarta+ (39) databases of known NEMGs, 50 of the total T2D *cis-*genes were defined as NEMGs, associated with 40 independent loci. These 50 T2D *cis-*NEMGs are listed in **Table 1** with the corresponding T2D (*Ŝ_T2D_*) and eQTL (*Ŝ_eQTL_*) location estimates (b37) obtained from LDU-based gene mapping. Of these 40 loci, 20 have been independently associated with T2D in two single-SNP GWAS studies: 13 by Mahajan et al. (2018) (1) and an addition seven in the recent GWAS by Vujkovic et al. (2020) (2) (based on the replication criteria of Vujkovic et al. requiring lead SNPs to be within 500kb, here defined as the distance between lead GWAS SNPs and *Ŝ_T2D_* locations).

**Table 1:** T2D and eQTL location estimates (physical coordinate, b37) associated with 50 T2D *cis-*NEMGs.

T2D locations p-value < 10^-5^						T2D locations p-value < 10^-5^					
Chr	T2D location GWAS-E^a^	T2D location GWAS-A^b^	T2D location metabo-E^c^	*Cis-*NEMG	eQTL location^d^	Chr	T2D location GWAS-E^a^	T2D location GWAS-A^b^	T2D location metabo-E^c^	*Cis-*NEMG	eQTL location^d^
1	26006625	–	26003695	*MTFR1L*	25900571	12	121317223	–	121243696	*ACADS*	121372772
1	26006625	–	26003695	*CLIC4*	26002616	12	121317223	–	121243696	*GATC*	121186959
1	26006625	–	26003695	*CLIC4*	25900772	13	102408534	102452781	N/A	*PCCA*	102428282
1	234270220	234273988	N/A	*COA6*	234338743	13	111049674	111004953	111035483	*NAXD*	111060677
1	234270220	234273988	N/A	*TOMM20*	234309446	15	–	63345547	63425768	*LACTB*	63453484
2	–	204023399	203970861	*NIF3L1*	203798637	20	–	25769672	25727136	*ACSS1*	25740998
2	–	204023399	203970861	*FLJ38973*	204126463	22	33046025	33046036	N/A	*PISD*	33058307
2	–	204023399	203970861	*MARS2*	203820504	**T2D locations p-value < 10^-3^**					
2	–	204023399	203970861	*CPS1*	204126514	2	–	194739106	194690969	*COQ10B*	194842026
2	227080369	–	227021099	*MFF*	227151692	2	–	194739106	194690969	*HSPD1*	194213959
3	67744088	67685265	N/A	*SLC25A26*	67577196	2	200305909	–	200330147	*FLJ38973*	200457932
3	123048537	–	123061689	*CCDC58*	122876198	2	200305909	–	200330147	*MAIP1*	200070891
3	120573472	120555505	N/A	*NDUFB4*	120573615	6	–	76217291	76199095	*COX7A2*	76466553
3	132436519	–	132429438	*ACAD11*	132451038	7	48732315	48812202	N/A	*ABCA13*	48628852
3	183260285	183210822	N/A	*MCCC1*	183260250	7	140349757	140367908	N/A	*MRPS33*	140378682
4	91942692	91950656	N/A	*PDHA2*	91947875	10	112924900	112866891	–	*GPAM*	112917454
4	104004185	–	103936988	*CISD2*	104141231	11	61284211	61258729	N/A	*FEN1*	61260634
6	112808197	112750188	N/A	*GPAM*	112800751	12	12633435	12621259	N/A	*HEBP1*	12634131
6	127539286	–	127502744	*TRMT11*	127479024	12	123387213	123750895	123447928	*DIABLO*	123386662
6	127539286	–	127502744	*HINT3*	127357421	12	123387213	123750895	123447928	*DIABLO*	123386489
10	94499812	–	94479016	*Mar-05*	94488072	12	123387213	123750895	123447928	*ABCB9*	123260234
10	104786704	–	104841790	*SFXN2*	104732175	12	123387213	123750895	123447928	*ABCB9*	123386756
11	8551677	–	8637191	*CYB5R2*	8667032	12	123387213	123750895	123447928	*ABCB9*	123386769
11	43879353	–	43879882	*ALKBH3*	43607412	12	123387213	123750895	123447928	*COXPD7*	123439939
11	65575917	–	65600493	*MRPL11*	65466334	12	133105848	–	133168320	*PGAM5*	133182753
12	56618300	–	55152275	*GLS2*	56628724	15	77270791	–	77310648	*IDH3A*	77085108
12	56618300	–	55152275	*SUOX*	56621500	16	9759261	–	9794698	*ABAT*	9799479
12	112991642	–	112988341	*ALDH2*	112229375	21	44327412	44352930	N/A	*NDUFV3*	44210786
12	106406604	106384733	N/A	*MTERF2*	106410234						

All eQTL locations (Ŝ_eQTL_) are within ±1LDU of a T2D location estimate (Ŝ_T2D_) for the ^a^European WTCCC1 cohort (GWAS-E), ^b^African American NIDDK cohort (GWAS-A) and ^c^European WTCCC2 MetaBoChip study cohort (GWAS metabo-E) (signals with low SNP coverage, indicated by N/A, were not analyzed). ^d^Ŝ_eQTL_ were generated using subcutaneous adipose gene expression for a population-based European sample from the MuTHER consortium.

### *Cis*-NEMGs Are Differentially Expressed by T2D Status

The hypothesis that the reported *cis-*genes are regulated by genetic variants associated with T2D was further explored by comparing the levels of gene expression using independent cohorts of T2D cases and controls. A total of 12 independent gene expression datasets for T2D or insulin resistant (IR) cases and controls were retrieved from the public GEO repository after the application of strict inclusion and exclusion criteria, listed in **Supplementary Table S2**. Expression datasets were included for adipose, muscle, liver and pancreas, in order to explore potential effects across multiple T2D-relevant tissues. The final 12 datasets are listed in **Supplementary Table S3** and extended information is provided in **Supplementary Table S4**. One dataset provided data for both skeletal muscle and subcutaneous adipose (accession: GSE13070). Another skeletal muscle dataset (accession: GSE25462) included gene expression data for normoglycemic individuals with zero, one or two T2D-affected parents, which provided an opportunity to assess informative changes in gene expression in individuals without overt T2D, but who may have a greater genetic susceptibility for T2D risk due to their positive family history.

The T2D *cis-*genes and *cis-*NEMGs were tested for enrichment of differential expression in T2D or IR cases using gene set enrichment analysis (GSEA) in the meta-analyzed datasets, grouped by tissue type. GSEA offers a powerful means to detect consistent patterns of differential expression within a set of related genes (23, 58). In this instance, the sets of total T2D *cis-*genes (n = 763) and *cis-*NEMGs (n = 50) were tested for differential expression using a competitive GSEA and gene-centric Z scores as summary measures of differential gene expression. Four GSEA analyses were carried out: the first two tests compared (1) the T2D *cis-*gene and (2) the *cis-*NEMG gene sets against the genomic background. The third (3) tested the T2D *cis-*NEMGs compared to the background of all NEMGs. The motivation for the third comparison was to test whether the 50 T2D *cis-*NEMGs showed additional differential expression compared to the background of all known NEMGs, which are generally down-regulated in individuals with T2D (data not shown). This comparison aimed to detect any evidence consistent with the alternative hypothesis that genetic mechanisms may dysregulate key NEMGs in T2D, compared with a null hypothesis that NEMGs may show non-specific down-regulation as a consequence of T2D onset. The fourth test (4) provided a negative control for this comparison and compared three control sets of randomly selected NEMGs (excluding the identified *cis-*NEMGs and therefore referred to as ‘non-T2D’ NEMGs in this study) to the NEMG background.

The results of the GSEA are shown in **Table 2** for the meta-analyzed datasets (summary GSEA results for each individual datasets are provided in **Supplementary Table S5**). Firstly, the total 763 T2D *cis*-genes were significantly enriched for decreased expression in T2D and IR cases compared to controls in skeletal muscle, liver and pancreas (see **Table 2A**) and were enriched for both increased and decreased (mixed) differential expression in adipose. The T2D *cis-*genes were also enriched for decreased expression in the skeletal muscle of unaffected individuals with an increasing number of parents affected by T2D, demonstrating a detectable change in gene expression in the absence of overt disease onset. For the subset of 50 *cis-*NEMGs, GSEA confirmed significant down-regulation in cases in three out of the four tissues (**Table 2B**), as well as the in the skeletal muscle of normoglycemic individuals with affected parents. Liver was not significant, however this tissue notably had the smallest combined sample size with only 20 cases.

**Table 2:** *Cis*-gene expression in Type 2 diabetes (T2D) and insulin resistant (IR) cases **vs** controls.

Tissue (# of datasets)	(A) T2D *cis-*genes (n=763) *vs* genomic background	(B) T2D *cis-*NEMGs (n=50) *vs* genomic background	(C) T2D *cis-*NEMGs (n=50) *vs* all NEMGs	(D) Random NEMGs (n=50) *vs* all NEMGS (three control sets)		
Adipose (n=5)	4.2E-03 (4.2E-03)	↓7.0E-04 (1.4E-03)	0.03 (0.11)	0.21 (n.s.)	↑0.03 (0.15)	0.14 (n.s.)
Muscle (n=3)	↓1.4E-03 (1.4E-03)	↓<1.0E-04 (2.0E-04)	0.079 (n.s.)	0.078 (n.s.)	0.24 (n.s.)	0.53 (n.s.)
Liver (n=2)	↓6.6E-03 (0.01)	0.11 (n.s.)	0.30 (n.s.)	0.46 (n.s.)	0.37 (n.s.)	0.13 (n.s.)
Pancreas (n=3)	↓2.8E-03 (2.8E-03)	↓2.0E-04 (4.0E-04)	↓0.01 (0.04)	0.057 (n.s.)	0.57 (n.s.)	0.87 (n.s.)
Skeletal muscle (FH) (n=1)	↓0.02 (0.02)	↓3.0E-04 (6.0E-04)	↓0.04 (0.16)	0.81 (n.s)	0.29 (n.s.)	0.08 (n.s.)

Meta-analyzed datasets for subcutaneous adipose, skeletal muscle, liver and pancreas were used for gene set enrichment analysis (GSEA) to test differential gene expression in T2D and IR cases for **(A)** the T2D cis-genes and **(B)** cis-NEMGs against the genomic background, as well as **(C)** the cis-NEMGs and **(D)** three sets of random NEMGs against the background of all known NEMGs (n=1,204 from the combined Mitocarta2.0/+). P-values are presented with false discovery rates (FDR) that account for multiple-testing in brackets. ↓ indicates a significant enrichment for decreased expression compared to the background and no arrow indicates an enrichment for mixed differential expression (both increased and decreased). An additional dataset with family history (FH) data shows differential expression in unaffected individuals depending on the number of parents with T2D. n.s., not significant (p-value > 0.05). ↑indicates a significant enrichment for increased expression in one of the 15 permuted control datasets.

If mitochondrial dysfunction were exclusively a consequence of T2D onset, then the *a priori* expectation would be for NEMGs to show generalized differential expression for individuals with T2D or IR, regardless of inferred *cis-*NEMG association with T2D genetic risk loci or not. Indeed, the set of total NEMGs in Mitocarta2.0/+ were generally downregulated across all datasets (data not shown). Here however, we demonstrate that this effect is not just a general feature of T2D, but can also be stratified by disease status for *cis-*NEMGs that are associated with T2D-eQTL and NEMGs that are not. GSEA provided evidence that the 50 T2D *cis-*NEMGs were enriched for differential expression compared to the background of all NEMGs (n = 1,204) (see **Table 2C**) for the meta-analyzed adipose (p-value = 0.03) and pancreas tissues (p-value = 0.01), as well as in skeletal muscle for normoglycemic individuals with an increasing number of parents affected with T2D (p-value = 0.04). Only pancreatic tissue was significant after correction by FDR (FDR = 0.04), although the GSEA power is likely to be limited by the high correlation structure between NEMG expression. Three negative control sets of 50 random NEMGs showed no significant differences in expression compared to the NEMG background, excluding one nominal result from the total 15 tests (see **Table 2D**). This result was also observed in the normoglycemic offspring of individuals with affected parents, demonstrating changes in this subset of NEMGs compared to other NEMGs even in the absence of overt disease.

### NEMG Expression in T2D: *Cis-*NEMGs Compared to Other NEMGs

**Figure 3:**
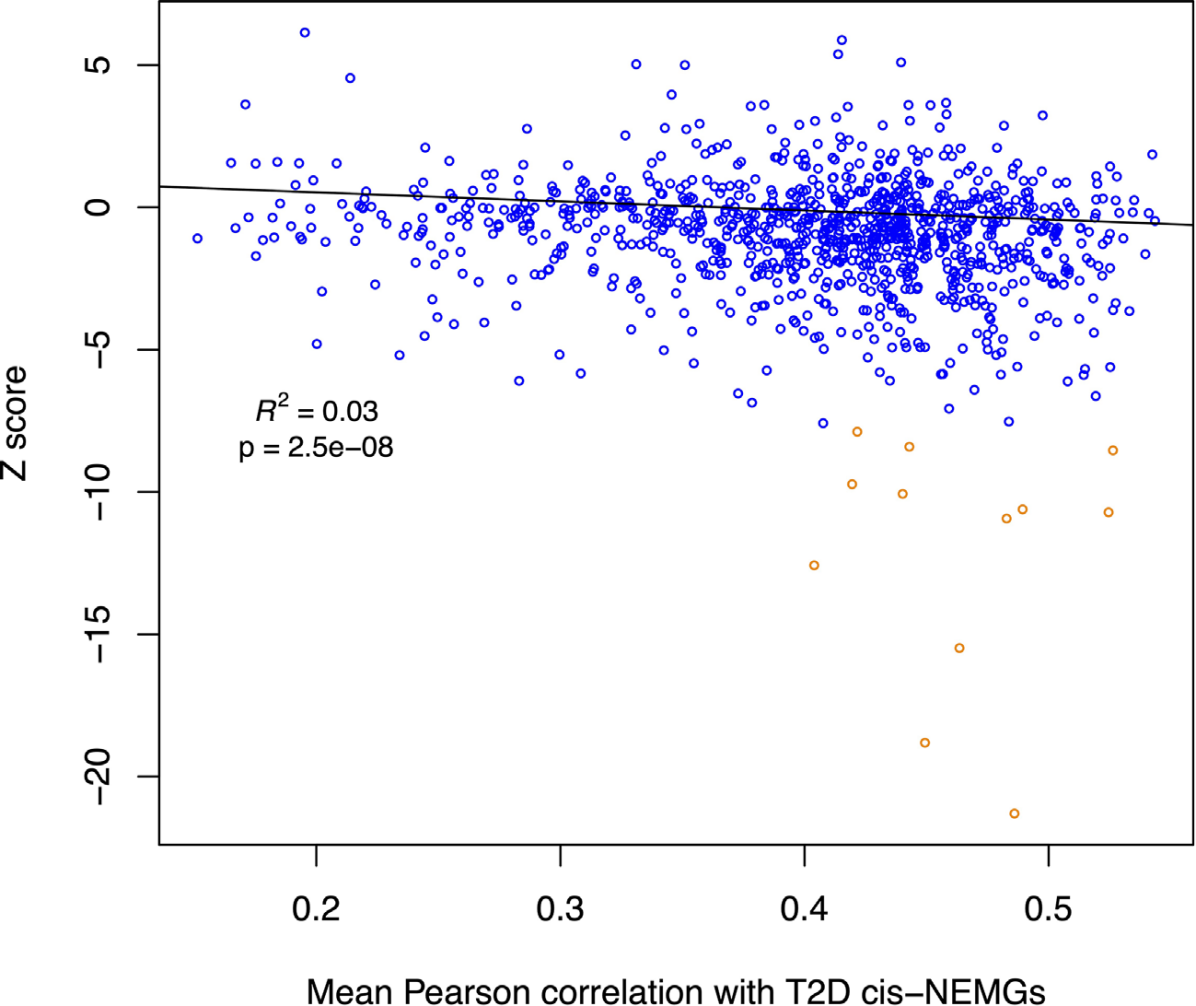
Scatterplot for non-T2D NEMG subcutaneous adipose differential expression by degree of correlation with T2D *cis*-NEMGs. The summary statistic of meta-analyzed adipose differential expression in T2D and IR cases (Z-score) for each non-T2D NEMG (n = 1,155) is plotted against its mean Pearson pairwise correlation coefficient for the T2D *cis-*NEMGs (n = 50), with the latter based on baseline expression levels in control individuals. NEMGs in general show reduced gene expression in cases compared to controls if they are more highly correlated with the 50 T2D *cis-*NEMGs (regression p-value = 2.5E-08, adjusted for absolute gene expression levels). The fitted line for the regression excludes 12 outlying data points (dark orange in the scatterplot) identified using the iterative robust regression procedure *rreg* implemented in Stata.

An additional analysis was carried out to further investigate NEMG expression in T2D and IR. The differential expression levels of all NEMGs (summary Z scores) excluding the 50 T2D *cis-*genes (n = 1,155, referred to as ‘non-T2D’ NEMGs) were assessed for correlation with the 50 T2D *cis-*NEMGs in normoglycemic (control) individuals (n=11, GSE27949). Using linear regression including average gene expression levels as a covariate, a significant negative relationship was observed between the Z score (case *vs* control differential expression) in adipose tissue of these 1,155 non-T2D NEMGs and their mean pairwise correlation with the 50 T2D *cis-*NEMGs (p-value = 2.5E-08). **Figure 3** illustrates the decrease in gene expression in T2D cases for NEMGs which correlated more highly with the 50 T2D *cis-*NEMGs. This analysis effectively differentiates changes in NEMG expression based on the expression levels of the subset of 50 T2D *cis-*NEMGs identified by *Ŝ_T2D_*-*Ŝ_eQTL_* co-location. Following the identification of these candidate genes, it will be of future interest to investigate the consequences of their altered expression and the potential for driving the wider changes observed in highly correlated NEMGs.

### *Cis*-NEMG Functions and Enrichment of Mitochondrial Pathways

**Figure 4:**
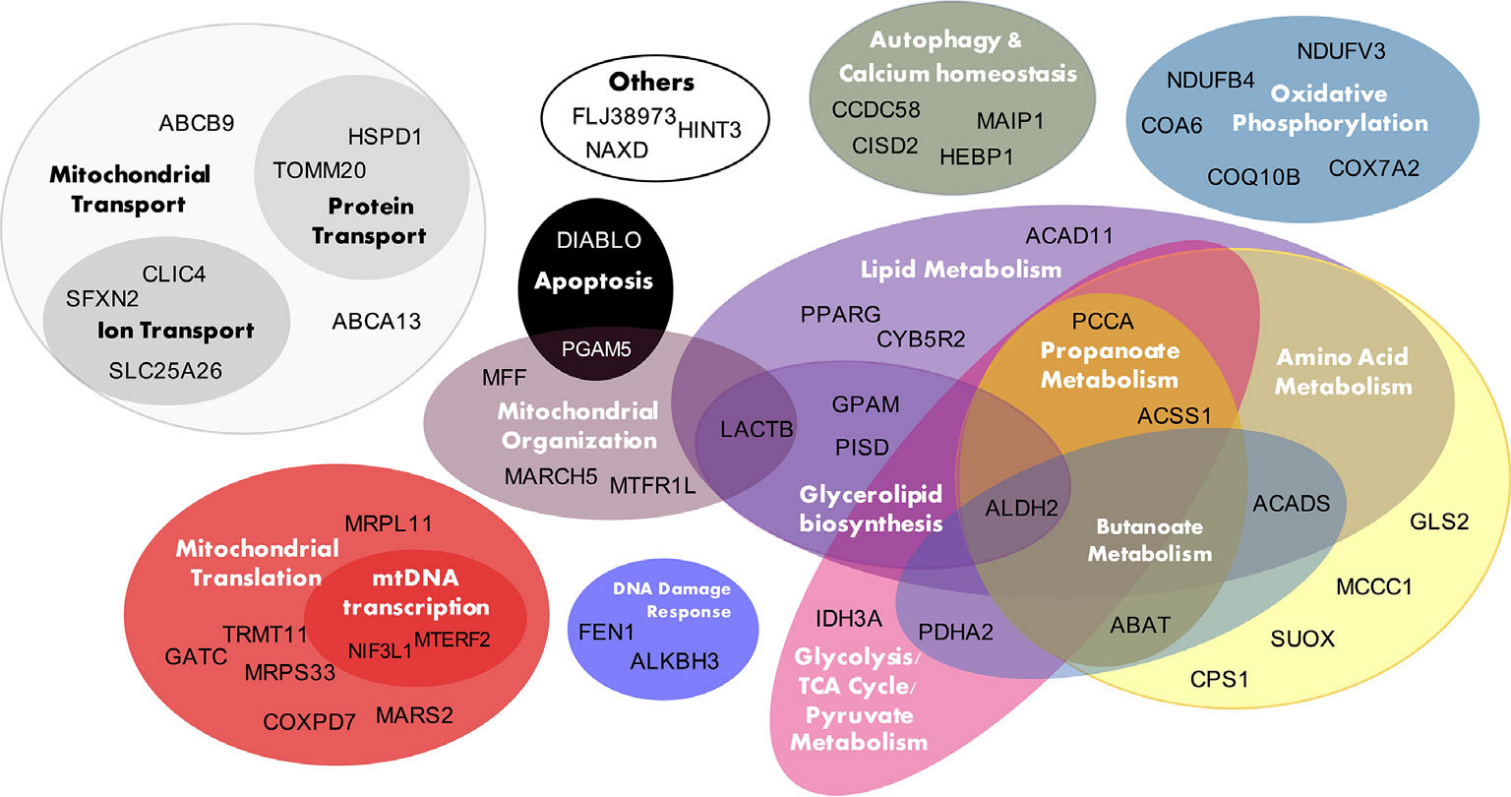
T2D *cis-*NEMG biological function. The 50 T2D *cis*-regulated nuclear encoded mitochondrial genes (*cis*-NEMGs) are grouped by common biological pathways (see **Supplementary Table S1** for extended information).

**Figure 4** presents the 50 T2D *cis*-NEMGs grouped by common biological functions and pathways, with a full summary list provided in **Supplementary Table S1** (**Supplementary Table S1** also cites evidence from the literature for a subset of these 50 NEMGs which have been previously linked to diabetes). These groups descriptively suggest biological pathways involved in T2D etiology, the largest of which include lipid and amino acid metabolism and suggest a heritable susceptibility to dysregulated mitochondrial metabolism in T2D adipose tissue. Of particular interest are *cis*-NEMGs which catalyze nearby steps on the same pathway, most notably branched chain amino acid (BCAA) catabolism with five *cis*-NEMGs: *PCCA*, *MCCC1*, *ABAT*, *ACADS* and *ALDH2* (**Supplementary Figure S3**). GSEA indicated that T2D/IR cases had decreased expression of genes involved in BCAA catabolism, supporting mounting evidence for a role of this pathway in T2D risk. While NEMGs were not generally enriched in the total count of T2D *cis*-genes compared to the genomic background - comprising 6.6% of the total compared to 5.4% of the genomic background (estimated using the total 21,215 genes annotated to the genomic IlluminaHT-12 v.3 BeadChip used for eQTL mapping) (Fisher-exact p-value = 0.20) - several mitochondrial pathways were enriched. Of 41 pre-defined gene sets relating to mitochondrial pathways, obtained from the curated Molecular Signatures Database (MSigDB) (56, 57), four gene sets showed nominal evidence of enrichment; these are shown in **Table 3** and include valine, leucine and isoleucine (branched chain amino acid, BCAA) catabolism, biotin-dependent carboxylases, propanoate and butanoate metabolism. The gene sets have significant overlap, with the two biotin-dependent carboxylases (*PCCA* and *MCCC1*) present in the BCAA gene set and *PCCA* also contributing to propanoate metabolism. *Cis*-genes in the BCAA catabolism gene set also overlapped with propanoate metabolism (*ABAT*, *ACSS1*, *PCCA* and *ALDH2*) and butanoate metabolism (*ABAT*, *ACADS*, *PDHA2* and *ALDH2*).

**Table 3:** Four mitochondrial pathways enriched in the total T2D *cis-*genes.

MSigDB C2 Gene Set (Source)	(A) Gene set enrichment			(B) GSEA in meta-analyzed case *vs* control gene expression data			
	# *cis*-genes	p-value *(random)*	p-value *(structured)*	Skeletal Muscle	Adipose	Liver	Pancreas
BCAA degradation	5	0.020	0.027	↓<2.5E-04	↓1.7E-04	↓6.0E-03	↓2.5E-04
Biotin carboxylases	2	0.022	0.019	↓9.0E-03	↓2.5E-04	0.20 (n.s.)	↓7.5E-04
Propanoate metabolism	4	0.025	0.042	↓<2.5E-04	↓1.7E-04	0.13 (n.s.)**	↓3.3E-04
Butanoate metabolism	4	0.032	0.042	↓9.0E-03	↓1.7E-04	0.14 (n.s.)**	↓2.5E-04

**(A)** Four curated mitochondrial gene sets showed evidence of enrichment by count among the total 763 T2D cis-genes (p-value<0.05), compared to the genomic background. All gene sets were curated from the KEGG database excluding biotin carboxylases which was manually defined. Empirical p-values were calculated using a permutation approach by selecting genes at random from the genomic background (random) or from within ±1.5Mb of each other (structured) (the distance used to investigate putative cis-genes). **(B)** FDR values from GSEA for the same four gene sets, showing significant enrichment for decreased expression in meta-analyzed case-control gene expression datasets. ↓ indicates a significant enrichment for decreased expression compared to the background and no arrow indicates an enrichment for mixed differential expression (both increased and decreased). n.s., not significant (p-value > 0.05).

The four mitochondrial gene sets showing evidence of enrichment in the total T2D *cis-*genes were also tested for differential expression. In the case-control gene expression meta-analysis, BCAA metabolism was enriched for decreased expression in 4 out of 4 tissues; propanoate metabolism in 3 out of 4 tissues (muscle, pancreas, and adipose); biotin-dependent carboxylases in 3 out of 4 tissues (muscle, adipose and pancreas); and butanoate metabolism in 3 out of 4 tissues (muscle, adipose and pancreas) (FDR <0.05) (**Table 3B**). BCAA catabolism, propanoate and butanoate metabolism were also significantly downregulated in the skeletal muscle of normoglycemic individuals with affected parents.

### T2D *Cis*-NEMGs Include Both Novel and Known T2D-Related Genes and Pathways

The T2D *cis-*NEMGs include both novel genes, which have not been previously reported in the literature in relation to T2D, as well as genes with published links to diabetes. Approximately half of the *cis-*NEMGs have previously been linked to diabetes or IR and **Supplementary Table S1** cites functional evidence from the literature. We here provide the likely locations of T2D risk variants which may regulate the expression of these genes. Examples include *CISD2*, a causal gene for Wolfram syndrome which manifests with early-onset diabetes (59, 60) and *ABAT*, which encodes Gamma-aminobutyric acid transaminase (GABA-T). GABA-T knockdown improves insulin sensitivity in obese mice, causing weight loss and decreased food intake (61). As another example, the T2D *cis-*gene *GPAM* encodes glycerol-3-phosphate acyltransferase and its overexpression has been shown to induce IR (62–64). At a pathway level, the *cis-*NEMGs implicate T2D-related molecular pathways including BCAA catabolism (*cis-*genes: *PCCA*, *MCCC1*, *ACADS*, *ABAT* and *ALDH2*). BCAA levels demonstrate striking power as predictive biomarkers of T2D (65, 66) and BCAA catabolism is intricately entwined with metabolic homeostasis (67), insulin secretion, adipocyte differentiation (68, 69), energy homeostasis (70), inflammation (71, 72) and appetite (73). Similarly, there is a diverse literature linking fatty acid β-oxidation (*cis-*genes: *ACAD11, ACADS*) to T2D, including proposed mechanisms through which decreased oxidation of fatty acids and accumulation of intermediates may cause insulin resistance and T2D [reviewed in (74)]. Prolonged inhibition of β-oxidation in mice was recently shown to reduce insulin sensitivity and increase hepatic glucose production (75). In addition to metabolic pathways, the T2D *cis-*NEMGs may also implicate general mitochondrial function in T2D genetic risk, including mitochondrial transcription and translation (*MRPL11*, *MRPS33*, *TRMT11*, *COXPD7*, *GATC*, *MARS2*); fission (*MARCH5*, *MFF*, *MTFR1L*); organization and dynamics (*LACTB*, *PGAM5*); transport (*SLC25A26*, *CLIC4*, *SFXN2*, *TOMM20*, *HSPD1*, *ABCA13*, *ABCB9*) and the electron transport chain (*COQ10B*, *NDUFB4*, *COX7A2*, *NDUFV3* and *COA6*).

## Discussion

This study investigated evidence of genetic mechanisms which may contribute to mitochondrial dysfunction in T2D. LDU-based gene mapping provided evidence of 50 NEMGs which may be *cis-*genes regulated by genetic variants associated with an increased risk of T2D. This approach yielded three novel outcomes. Firstly, *Ŝ_T2D_*-*Ŝ_eQTL_* co-location within 1 LDU identified disease-relevant *cis*-genes which showed independent evidence of differential expression in individuals with T2D, IR and a positive family history of T2D, compared to controls (see **Table 2**); this result supports the use of eQTL mapping in identifying the likely target genes of T2D loci. Secondly, *Ŝ_T2D_*-*Ŝ_eQTL_* co-location identified 50 *cis-*NEMGs which showed differential expression in cases to a greater extent than other NEMGs, with NEMGs not identified as T2D *cis-*genes in turn also showing reduced expression if their expression correlated more highly with the subset of 50 *cis-*NEMGs. This differentiation of general NEMG expression raises the possibility that genotype-dependent changes in key NEMGs may drive wider perturbations in mitochondrial function and is worthy of further investigation. These results suggest that mitochondrial dysfunction may not exclusively be a consequence of T2D and instead may also support the role of heritable genetic variation in contributing to mitochondrial dysfunction prior to disease onset. Thirdly, four mitochondrial gene sets were enriched for T2D *cis-*genes: BCAA catabolism, biotin-dependent carboxylases, propanoate and butanoate metabolism. Overall, these three outcomes support our conclusion that genetic mechanisms may contribute to mitochondrial dysfunction in T2D.

One subsidiary aim of this study was to provide additional validation that LDU-based gene mapping can successfully map disease-eQTL and functional *cis-*genes. Independent evidence was used to demonstrate that the mapped *cis-*genes were differentially expressed in individuals with T2D and IR. This is in line with recent *in vivo* evidence that a novel T2D *cis-*gene previously mapped using the same method (76), *ABCC5*, was functionally implicated in diabetes by improving insulin sensitivity and reducing fat mass when knocked-out in mice (76, 77). Association mapping using genetic LDU maps, as described by Maniatis et al. (4), offers significant power to identify disease-eQTL and associated *cis-*genes, by testing for evidence of association based on the decline of trait-association for genotyped variants in decreasing LD with a causal variant (see **Figure 1**). This mapping method can be applied to other complex diseases, including T2D subgroups which show distinct genetic associations (78–80).

A key assumption of this study is that co-locating *Ŝ_T2D_*-*Ŝ_eQTL_* capture shared causal variants. This is generally supported by the observed differential expression of the associated *cis*-genes in individuals with T2D or IR, but may also be addressed locus-by-locus. Functional studies will be necessary to confirm the causal *cis-*gene(s), since it is possible that neighboring genes may be co-regulated with a causal gene despite it not directly contributing to the T2D phenotype. Future LDU-based mapping of eQTL for other tissues will also offer wider insights into the role of NEMG regulation in T2D. Nevertheless, the current study provides important evidence regarding the role of adipose tissue mitochondrial function in inherited risk of T2D. These results implicate adipose tissue mitochondrial metabolism in T2D risk, particularly BCAA catabolism, biotin carboxylases, propanoate and butanoate metabolism (**Table 3A**), although additional pathways may be revealed by expanding the search to proteins such as nuclear transcription factors which do not localize to the mitochondrial proteome. Examples include *PPARG* and *SPATA18*, both of which were identified as T2D *cis-*genes but not NEMGs and play important roles in regulating mitochondrial function and quality, respectively.

Finally, these results may be of clinical interest, since many T2D drugs affect mitochondrial function (5) and NEMG regulation may impact treatment efficacy. For example, *MCCC1* and *PCCA* make up two of the five human biotin-dependent carboxylases. The efficacy of biotin, which may improve insulin sensitivity (81, 82), may be influenced by the dysregulation of these genes. The expression of GABA-transaminase (*ABAT*) may also influence the proposed therapeutic benefits of GABA treatment, which is currently undergoing clinical investigation. In conclusion, this study identified 50 NEMGs which may be effector genes of genotype-driven mitochondrial dysfunction in T2D and highlights key genes and pathways which may contribute to insulin resistance in adipose tissue. The methods described in this paper can be used to systematically interpret *cis*-gene functions to implicate novel biological pathways in the etiology of T2D and other complex traits.

## Data Availability Statement

The original contributions presented in the study are included in the article/**Supplementary Material**. Further inquiries can be directed to the corresponding author.

## Ethics Statement

All analyses use publicly available data. The patients/participants provided their written informed consent as part of each study data sample collection.

## Author Contributions

TA conceptualized the study with contribution from NM. NM, WL, and TA carried out initial data analysis and provided the data used in this study. HM drafted the MS and implemented the analyses and methods described. All authors contributed to the article and approved the submitted version.

## Funding

HM and TA would like to acknowledge the Medical Research Council, United Kingdom, for financially supporting this work (MRC DTP PhD Studentship and Investigator Award 91993, respectively). WL and NM would like to acknowledge the Wellcome Trust, United Kingdom, for financial support (Seed Award 209106/Z/17/Z).

## Conflict of Interest

The authors declare that the research was conducted in the absence of any commercial or financial relationships that could be construed as a potential conflict of interest.

## Publisher’s Note

All claims expressed in this article are solely those of the authors and do not necessarily represent those of their affiliated organizations, or those of the publisher, the editors and the reviewers. Any product that may be evaluated in this article, or claim that may be made by its manufacturer, is not guaranteed or endorsed by the publisher.
