# Supplementary material for: New Insights Into Mitochondrial Dysfunction at Disease Susceptibility Loci in the Development of Type 2 Diabetes
Source: Front Endocrinol (Lausanne). 2021 Aug 11;12:694893. doi: 10.3389/fendo.2021.694893 (PMC8385132; doi:10.3389/fendo.2021.694893)
Supplement: Supplementary file 1 [file DataSheet_1.pdf]

Table of Contents

Supplementary Table S1. T2D *cis*-NEMG functions. 2
Supplementary Table S2. GEO gene expression datasets inclusion and exclusion criteria. 6
Supplementary Table S3. GEO gene expression datasets. 7
Supplementary Table S4. Summary information for all GEO gene expression datasets analysed............9
Supplementary Table S5. Gene set enrichment analysis for all GEO datasets............13
Supplementary Table S6. All mitochondrial pathways tested for enrichment. 14

Supplementary Figure S1. GEO gene expression dataset selection 7
Supplementary Figure S2. GEO dataset correlations 11
Supplementary Figure S3. Five T2D *cis*-NEMGs on the branched chain amino acid catabolism (BCAA) pathway............12

Supplementary Table S1. T2D *cis*-NEMG functions. The 50 *cis*-NEMGs are sorted into functional groups. A brief summary of protein function is provided, with the relevant KEGG[1] pathways, KEGG ontology terms and/or Genecards summaries (www.genecards.org)[2] presented. Colour-coded circles correspond to Figure 2 (main text). The fourth column includes citations and brief explanations for any genes which have been previously linked with diabetes in the literature. This list aims to identify all relevant publications but may not be exhaustive.

- Amino acid metabolism
- Butanoate metabolism
- Propanoate metabolism
- Mitochondrial transport
- Lipid metabolism
- TCA cycle / glycolysis
- Autophagy & calcium homeostasis
- Oxidative phosphorylation
- Mitochondrial translation
- Mitochondrial transcription
- Protein / ion transport
- Mitochondrial organization
- Apoptosis
- DNA damage response

Gene	Summary of protein function (KO=KEGG ontology)	Group	Evidence linking *cis*-gene to diabetes in the literature
ABAT	**4-Aminobutyrate aminotransferase**. Encodes Gamma-aminobutyric acid transaminase (GABA-T). **KEGG pathways:** alanine, aspartate and glutamate metabolism; valine, leucine and isoleucine (BCAA) degradation; beta-Alanine metabolism; propanoate metabolism; butanoate metabolism	Amino acid metabolism; Propanoate metabolism; Butanoate metabolism	Hepatic ABAT expression is positively associated with plasma insulin and negatively with insulin sensitivity (IS) in humans[3]. GABA-T function is reduced in Diabetic rat retinas[4]. GABA-T knockdown improves IS in obese mice, causing weight loss and decreased food intake[3]. GABA stimulates β-cell replication, enhances insulin secretion, protects against apoptosis and suppresses glucagon secretion[5-11]. GABA-treated high-fat diet (HFD)-mice show ↑glucose tolerance, ↑IS, ↓inflammation and ↑T_{reg}[6].
ABCA13	**ATP Binding Casette Subfamily A Member 13.** KEGG pathways: ABC transporters	Mitochondrial transport	-
ABCB9	KEGG pathways: ABC transporters; Lysosome	Mitochondrial transport	Locus associated with T2D[12; 13]. *ABCB9* implicated by eQTL analysis[14].
ACAD11	**Acyl-CoA Dehydrogenase Family Member 11**. KO: lipid metabolism. Genecards: "Acyl-CoA dehydrogenase, that exhibits maximal activity towards saturated C22-CoA"	Lipid metabolism	Polymorphisms in the *ACAD11* paralog, *ACAD10*, have been associated with T2D[15].
ACADS	**Acyl-CoA dehydrogenase short chain**. KEGG pathways: Fatty acid degradation; BCAA degradation; beta-alanine, propanoate, butanoate, carbon and fatty metabolism.	Lipid metabolism; Amino acid metabolism; Propanoate metabolism; Butanoate metabolism	ACADS-deficient mice were resistant to HFD-induced obesity and insulin resistance (IR)[16; 17]. Polymorphism associated with reduced glucose-stimulated insulin release[18].
ACSS1	**Acyl-CoA synthetase short chain family member 1.** KEGG pathways: Glycolysis / gluconeogenesis; pyruvate metabolism; glyoxylate and dicarboxylate metabolism; propanoate metabolism. KEGG Orthology: lipid metabolism.	Lipid metabolism; Propanoate metabolism; TCA cycle / glycolysis	-
ALDH2	**Aldehyde dehydrogenase.** KEGG pathways: fatty acid degradation; arginine and proline metabolism; BCAA degradation; histidine metabolism; tryptophan metabolism; beta-alanine metabolism; glycerolipid metabolism; pyruvate metabolism	Lipid metabolism; Amino acid metabolism; TCA cycle / glycolysis	May play a role in preventing high-glucose induced cellular dysfunction[19]. Coding mutation associated with T2D and fasting blood glucose[20-22]. May protect against Diabetic cardiomyopathy[23].

ALKBH3	**Alpha-ketoglutarate dependent dioxygenase.** KO: DNA repair and recombination proteins.	-
CCDC58	**Coiled-coil domain containing 58.**	-
CISD2	**CDGSH iron sulfur domain 2.** Genecards: "Regulatory of autophagy… required for BCL2-mediated depression of endoplasmic reticulum [calcium] stores during autophagy."	Mutations cause Wolfran syndrome, which manifests with early-onset diabetes[24-27].
CLIC4	**Chloride intracellular channel protein 4**. KO: ion channels.	Sensitises pancreatic β-cells to apoptosis[28].
COA6	**Cytochrome c oxidase assembly factor 6.** KEGG pathways: thermogenesis. KO: mitochondrial respiratory chain complex assembly factors / complex-IV assembly factors	-
COX7A2	**Cytochrome c oxidase subunit 7a.** KEGG pathways: oxidative phosphorylation; thermogenesis	-
COXPD7	**Chr12 open reading frame 65.** KO: mitochondrial biogenesis / mitochondrial transcription and translation factors.	-
COQ10B	**Coenzyme Q-binding protein.** KO: COQ10.	CoQ10 treatment in Diabetic rat models improve metabolic measures[29; 30] and protects against Diabetic nephropathy[31; 32]. CoQ10 supplementation improves HbA1C levels in individuals with diabetes[33].
CPS1	**Carbamoyl-phosphate synthase 1.** KEGG pathways: arginine biosynthesis; alanine, aspartate and glutamate metabolism; biosynthesis of amino acids.	Genetic variance associated with lower risk of T2D and altered glycine metabolite levels[34]. SNP associated with BMI and directionally consistent with T2D association[35-37].
CYB5R2	**Cytochrome-b5 reductase.** KEGG pathways: amino sugar and nucleotide sugar metabolism.	-
DIABLO	**DIABLO**. KEGG pathways: Apoptosis.	-
FEN1	**Flap endonuclease 1**. KEGG pathways: DNA replication; base excision repair; non-homologous end-joining.	-
FLJ38973	**Chr2 open reading frame 69.**	-
GATC	**Aspartyl-tRNA(Asn)/glutamyl-tRNA(Gln) aminotransferase subunit C.** KEGG pathways: aminoacyl-tRNA biosynthesis. KO: mitochondrial transcription and translation factors.	SNPs associated with diabetic kidney disease in Type 1 diabetes patients[38].
GLS2	**Glutaminase 2**. KEGG pathways: arginine biosynthesis; alanine, aspartate and glutamate metabolism; D-glutamine and D-glutamate metabolism.	*GLS2* missense mutations cause increased glucose production, while mouse knockouts cause lower blood glucose levels in insulin resistant conditions[39]. Genetic variants associated with higher fasting plasma glucose[40; 41].
GPAM	**Glycerol-3-phosphate acyltransferase, mitochondrial.** KEGG pathways: glycerolipid metabolism; glycerophospholipid metabolism.	Knockout mice show hyperinsulinemia and reduced glucose tolerance[42]. Overexpression causes IR[43-45] and knock-down improves IS[46].

HEBP1	**Nuclear factor, erythroid 2 like 2.** Genecards: “Promotes calcium mobilization and chemotaxis in monocytes and dendritic cells”.	-
HINT3	**Histidine triad nucleotide binding protein 3.**	-
HSPD1	**Heat shock protein family D (Hsp60) member 1.** KEGG pathways: RNA degradation; Type 1 diabetes mellitus. Genecards: “This protein is essential for the folding of newly imported proteins in the mitochondria.”	Tissue-specific alterations in Hsp60 levels in T2D[47; 48], which can be altered by hyperglyemic conditions[49]. Implicated in hyperglycemia-induced renal tubular dysfunction[50]. Dysregulated in T2D hypothalamus[51]. An important modulator of inflammation in autoimmune diabetes[52-55]. Immunity to the Hsp60 peptide p277 can cause diabetes in mice[56]. Hsp60 modulates adiponectin signalling[57].
IDH3A	**Isocitrate dehydrogenase 3 (NAD+) alpha.** KEGG pathways: citrate cycle (TCA cycle); biosynthesis of amino acids	Decreased expression in T2D skeletal muscle[58]. Knock-down in combination with IDH2 may perturb glucose-stimulated insulin secretion[59].
LACTB	**Serine beta-lactamase-like protein LACTB, mitochondrial.** KEGG pathways: none. KO: serine peptidases. Genecards: “Acts as a regulator of mitochondrial lipid metabolism... by decreasing protein levels of PISD…” [2]: forms stable filaments in the mitochondrial intermembrane space, promoting mitochondrial organization and micro-compartmentalization.	-
MARCH5	**MARCH5.** KEGG pathways: none. KO: RING-type E3 ubiquitin transferase. Genecards: “…plays a crucial role in the control of mitochondrial morphology by acting as a positive regulator of mitochondrial fission. May play a role in the prevention of cell senescence acting as a regulatory of mitochondrial quality control.”	-
MAIP1	**Matrix AAA peptidase interacting protein 1.** KEGG pathways: none. Genecards: “Promotes sorting of SMDT1 - a core regulatory component of a calcium channel in the mitochondrial inner membrane - in mitochondria by ensuring its maturation.”	-
MARS2	**Methionyl-tRNA synthetase.** KEGG pathways: aminoacyl-tRNA biosynthesis.	-
MCCC1	**Methylcrotonoyl-coA carboxylase subunit alpha, mitochondrial.** KEGG pathways: valine, leucine and isoleucine degradation.	Part of a gene network regulated proportionally with IS[60]. Down-regulated in IR adipose tissue[61; 62]. SIRT4 regulates IS in the pancreas via activation of MCCC1[63].
MFF	**Mitochondrial fission factor.** KEGG pathways: none. KO: mitochondrial dynamics / fission and fusion factors.	Excess MFF acts with NR4A1 to initiate mitochondrial fission and apoptosis in diabetic nephropathy[64]. MFF interacts with Mfi, which increases GSIS when knocked-down in mice[65].
MRPL11	**Mitochondrial ribosomal protein L11.**	-
MRPS33	**Mitochondrial ribosomal protein S33.**	*MRPS33* expression is altered and skeletal muscle IS is improved by *PLIN2* overexpression[66]. Differential methylation observed with gestational diabetes mellitus[67].

MTERF2	**Transcription termination factor, mitochondrial**. KO: mitochondrial transcription and translation factors.	-
MTFR1L	**Mitochondrial fission regulator 1 like.** Genecards: “An important paralog of this gene is MTFR2”	-
NAXD	**ATP-dependent NAD(P)H-hydrate dehydratase.** KEGG pathways: none. KO: hydro-lyases. Genecards: “Allows the repair of both epimers of NAD(P)HX, a damaged form of NAD(P)H”	-
NDUFB4	**NADH dehydrogenase (ubiquinone) 1 beta subcomplex subunit 4**. KEGG pathways: oxidative phosphorylation; thermogenesis	Expression is decreased in T2D muscle and adipose[68; 69]. Differentially expressed in mice with diabetic nephropathy[70]. Expression of the related *NDUFB6* correlates with IS[71].
NDUFV3	**NADH dehydrogenase (ubiquinone) flavoprotein 3.** KEGG pathways: oxidative phosphorylation; thermogenesis.	Altered expression in response to insulin[72]. Minor allele in *NDUFV3* associated with lower plasma glucose after oral glucose tolerance test[73].
NIF3L1	**NGG1 interacting factor 3 like 1.** Genecards: “May function as a transcriptional corepressor through its interaction with COP2.”	Interacts and forms a complex with WBSCR14, which is linked with Williams-Beuren syndrome (WBS) which manifests with impaired glucose tolerance and silent diabetes[74].
PCCA	**Propionyl-coA carboxylase alpha chain.** KEGG pathways: BCAA, propanoate, glyoxylate and dicarboxylate metabolism	Decreased expression in IR adipose[62].
PDHA2	**Pyruvate dehydrogenase E1 component alpha subunit.** KEGG pathways: glycolysis / gluconeogenesis; citrate cycle (TCA cycle); pyruvate metabolism	Knock-out of the paralogue PDHA1 in mouse islets impairs insulin secretion[75].
PGAM5	**PGAM family member 5, mitochondrial serine/threonine protein phosphatase.** KEGG pathways: mitophagy; necroptosis; TNF signaling	PGAM5 aggrevates development of diabetic renal tubular injury[76]. PGAM5-deficient mice are resistant to high-fat-diet-induced obesity and lipid accumulation[77].
PISD	**Phosphatidylserine decarboxylase.** KEGG pathways: glycerophospholipid metabolism.	-
SFXN2	**Sideroflexin 2.** KO: regulator of mitochondrial biogenesis; transporters; solute carrier family; protein involved in Fe-S cluster biogenesis.	Expression is associated with waist-hip ratio[78]. Gene expression increased in β-cell differentiation and in islets of diabetic rat models[79].
SLC25A26	**Solute carrier family 25.** KO: mitochondrial carrier. Genecards: “transports S-adenosylmethionine (SAM) into the mitochondria.”	-
SUOX	**Sulfite oxidase.** KEGG pathway: sulfur metabolism. Genecards: “This enzyme catalyses sulphite oxidation, the final reaction in the oxidative degradation of the sulfur amino acids cysteine and methionine.”	Dysreglated in the kidney of T1D mouse model[80]. Locus is associated with T1D and gene expression is associated with T1D risk variants[81-83].
TOMM20	**Mitochondrial import receptor subunit TOM20.** Genecards: “Central component of the receptor complex responsible for the recognition and translocation of cytosolically synthesized mitochondrial proteins.”	-
TRMT11	**tRNA (guanine19-N2)-methyltransferase.** KO: tRNA modifications.	-

Supplementary Methods

GEO gene expression datasets quality control and analyses.

Gene expression datasets

Datasets of T2D or insulin resistant (IR) case vs. control gene expression data for the tissues subcutaneous adipose, skeletal muscle, liver and pancreas were identified from the GEO database, using the search string:

(((((T2D OR Type 2 Diab* OR IGT OR insulin resistan* OR IFG OR pre*diab* OR impaired fasting glucose OR impaired glucose tolerance)) AND (homo sapiens[Organism] OR human[Organism])) AND cel[Supplementary Files]) AND expression profiling by array[DataSet Type]) AND (muscle OR skeletal muscle OR adipose OR adipo* OR omental OR subcutaneous OR skeletal muscle OR vastus lateralis OR rectus abdominus OR liver OR hepat* OR pancreas OR islet OR beta cell* OR myotub*)

Dataset suitability and quality control (QC)

In order to detect heritable changes in gene expression, strict inclusion and exclusion criteria, shown in Table S2, were applied to maximise homogeneity between datasets and reduce potential confounding of environmental effects or study design. In order to maintain consistency with our subcutaneous adipose eQTL design, datasets in which cases were treated with insulin were excluded in order to enrich for insulin resistant cases rather than insulin deficiency.

Supplementary Table S2. GEO gene expression datasets inclusion and exclusion criteria. *Pancreas datasets were exempt since samples were taken post-mortem and this information was not always available.

Inclusion criteria	Exclusion criteria
T2D or insulin resistant case-control study	Ongoing medications at the time of sampling*
Baseline gene expression measures	Non-European cohort
Raw data available as CEL files	Measurements from cultured cells
Data generated on an Affymetrix array	
Skeletal muscle, adipose, liver or pancreas	

Following preliminary filtering using the GEO search string above, dataset summaries were manually compared to the inclusion and exclusion criteria. Figure S1 shows the exclusion of datasets during this review stage. 26 datasets were subject to full text review, of which 13 were included in the final analysis; these are listed in Table S3. Detailed summary information regarding the study design and subjects for all datasets are presented in Table S4.

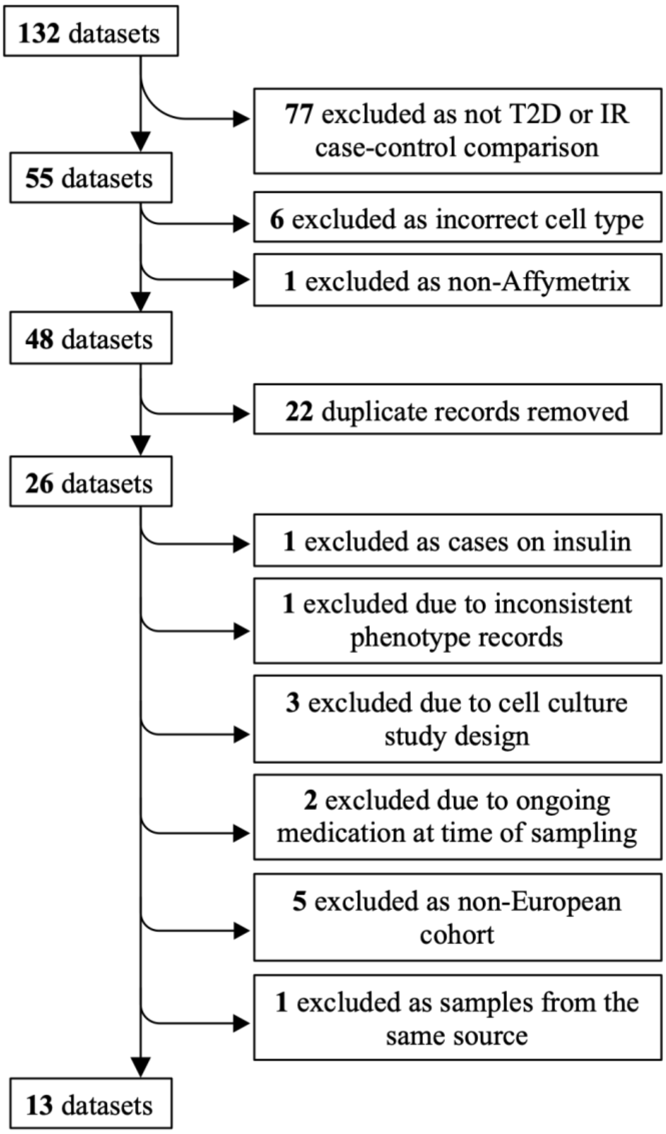

Supplementary Figure S1. GEO gene expression dataset selection. Gene expression datasets were excluded based on the inclusion and exclusion criteria listed in Table S2.

Supplementary Table S3. GEO gene expression datasets. Summary information for the final gene expression datasets obtained from GEO using the inclusion and exclusion criteria listed in Table S2. T2D = Type 2 diabetes, IR = insulin resistant. [a]Dataset GSE13070 provided data for both skeletal muscle and adipose. [b]Dataset GSE25462 also included information for unaffected, normoglycemic but IR offspring with one or two parents with T2D.

**GEO datasets included in meta-analysis**				
**GEO ID**	**Case/Control**	**Phenotype**	**Array**	**Tissue**
GSE13070[a]	51/18	IR	HG-U133_ Plus2	Skeletal muscle
GSE22435	10/7	T2D+IR	HG-U133_ Plus2	Skeletal muscle
GSE25462[b]	11/15	T2D	HG-U133_ Plus2	Skeletal muscle
GSE27949	10/11	IR	HG-U133_ Plus2	Subcutaneous adipose

GSE20950	9/10	IR	HG-U133_ Plus2	Subcutaneous adipose
GSE26637	5/5	IR	HG-U133_ Plus2	Subcutaneous adipose
GSE13070[a]	28/6	T2D	HG-U133_ Plus2	Subcutaneous adipose
GSE94753	18/21	IR	HuGene-1_1-st	Subcutaneous adipose
GSE15653	13/5	T2D	HG-U133A	Liver
GSE64998	7/8	T2D	HuGene-1_1-st	Liver
GSE76894	19/83	T2D	HG-U133_ Plus2	Pancreas
GSE25724	6/7	T2D	HG-U133A	Pancreas
GSE41762	20/57	T2D	HuGene-1_0-st	Pancreas

Microarray statistical analysis

All analyses were carried out using R version 3.6. For all datasets, raw CEL files were downloaded from GEO and corresponding R annotation packages were downloaded: Human Genome U133 Plus 2.0 Array, *hgu133plus2.db* v3.2.3 (21276 genes)[84]; Affymetrix Human Full Length HuGeneFL Array, *pd.hu6800* v3.2.3 (6021 genes)[85]; Affymetrix Human Gene 1.0 ST Array, *hugene10stprobeset.db* (20212 genes)[86]; Affymetrix Human Genome U95 Version 2 Array, *pd.hg.u95av2* (9667 genes)[87]. Raw data was normalized using Robust Multi-array Averaging (RMA) from the Bioconductor package, *oligo* v.1.48.0[88] and used to calculate gene-centric Z scores measuring differential expression between cases and controls, using a linear mixed-effects model to include multiple probes per gene as a random effect (in R, *lmer* from the *lme4* package for >1 probe or *lm* for 1 probe). Where available, age and BMI were included as covariates (see Table S4).

$$y = expression \sim caco + age + bmi + (1|probe)$$

Meta-analysis of gene sets was carried out according to Choi et al.[89] using the R package *GeneMeta* v.1.56.0.

For the purposes of meta-analysis, study design and dataset similarities were scrutinized according to the inclusion and exclusion criteria, resulting in the meta-analysis of datasets with the most consistent designs and lowest potential for confounding. Datasets in which cases were insulin treated were excluded in order to (1) enrich for insulin resistant individuals over insulin deficient, to be consistent with the study design of eQTL mapping in subcutaneous adipose (a tissue implicated in peripheral insulin resistance) and (2) reduce confounding of gene expression results due to insulin exposure. Inter-dataset correlations of gene level summary Z statistics (Z scores), representing case vs control expression on a global scale are presented in Figure S2. Global correlations were calculated using Z scores for genes with an absolute Z score >1 in both datasets in order to reduce non-significant noise.

Supplementary Table S4. Summary information for all GEO gene expression datasets analysed.

Skeletal Muscle

GEO ID, tissue & date submitted	Ca/Co (M/F)[a]	Array[b]	Ethnicity	Medication	Disease duration	Inclusion and Exclusion	Co-variates	BMI[c]	Age[c]	Fasting state	Family history
**GSE13070**[90] *Vastus lateralis* Oct 2008	NGT 18 T2D 51 **Mostly male*	HG-U133_Plus2	-	Discontinued two weeks prior.	-	Exclusion: Active cardiac, liver, or renal disease or long-term complications from diabetes.	-	NGT 24.6±0.8* T2D 35.5±0.8*	NGT 25±0.8* T2D 36±0.8*	-	-
**GSE22435**[91] *Rectus abdominus* Aug 2011	NGT 0/7 T2D 0/10	HG-U133_Plus2	Finnish	None	Diagnosed during the study (IGT=4 and T2D=3). No known glucose intolerance before.	Inclusion: receiving chole-cystectomy & post-menopausal. Normal liver, kidney and thyroid function, no history of excessive alcohol intake, and no major chronic illness.	Age & BMI	NGT 27.4±5.4 T2D 31.70±6.5	NGT 60±5.0 T2D 60±4.8	-	-
**GSE25462**[92] *Vastus lateralis* Mar 2011	FH- 7/8 FH+ 11/14 T2D 6/5	HG-U133_Plus2	-	None (educational interventions)	T2D subjects >1 year. FH+ individuals have NGT.	-	Age & BMI	NGT 25.1±3.3 1 parent 27.7±5.6 2 parents 28.4±6.9 T2D 32.80±8.1	NGT 38±12.0 1 parent 36±10.0 2 parents 40±12.0 T2D 50±14.0	Yes	Number of parents with T2D for controls.

Subcutaneous adipose

GEO ID, tissue & date submitted	Ca/Co (M/F)[a]	Array[b]	Ethnicity	Medication	Disease duration	Inclusion and Exclusion	Co-variates	BMI[c]	Age[c]	Fasting state	Family history
**GSE27949**[93] Mar 2011	NGT 11 IGT 10 T2D 12	HG-U133_Plus2	'Likely Scandin-avian'	Discontinued 24h or one week (hypo-glycemic) prior.	-	Exclusion: treatment with insulin, recent or ongoing infection, history of malignant disease or treatment with anti-inflammatory drugs.	Age & BMI	NGT 30.55±6.1 IGT 31.86±8.1 T2D 31.68±8.1	NGT 44±13.0 IGT 57±11.0 T2D 56±6.8	Yes	-
**GSE20950**[94] Mar 2010	NGT 2/8 IGT 4/5	HG-U133_Plus2	-	-	Patients diagnosed during the study.	Exclusion: T2D	-	NGT 48±3* IGT 49±7* *Matched*	NGT 39±6* IGT 43±9*	-	-
**GSE26637**[95] Jan 2011	NGT 0/5 IGT 0/5	HG-U133_Plus2	'Caucasian'	None	N/A	Inclusion: 18-60yrs; no known acute/chronic disease; BMI<40; not pregnant and not taking glucose tolerance-altering treatment.	-	NGT 22.0±0.7* IGT 32.5±1.7*	NGT 33±6* IGT 39±5*	-	-
**GSE13070**[90] Oct 2008	NGT 6 T2D 28	HG-U133_Plus2	-	Discontinued two weeks prior.	-	Exclusion: active cardiac, liver, or renal disease or long-term complications from diabetes.	-	NGT 24.6±0.8* T2D 35.5±0.8*	NGT 25±0.8* T2D 36±0.8*	-	-
**GSE94753**[96] Feb 2017	NGT 0/21	Hu	Residents of	2 contraceptive pills, 4 SSRI, 1	-	-	-	NGT 41±5* IGT 40±6*	NGT 36±7.8 IGT 40±8.1	Yes	

	IR 0/18	Gene-1_1-st	Stockholm, Sweden.	Duloxetine, 2 thiazide and amiloride. One IGT subject on multiple medications.						

Liver

GEO ID, tissue & date submitted	Ca/Co (M/F)[a]	Array[b]	Ethnicity	Medication	Disease duration	Inclusion and Exclusion	Co-variates	BMI[c]	Age[c]	Fasting state	Family history
**GSE15653**[97] Apr 2009	13/5	HG-U133A	-	None known	Diagnosed during the study	Inclusion: normal hepatic and thyroid function, no history of excessive alcohol intake, and no other chronic illness (undergoing gastric bypass surgery).	Age & BMI	NGT 51.5±4.4 T2D 52.4±6.6	NGT 39±10.9 T2D 46±12.4	Yes	-
**GSE64998**[98] Jan 2015	NGT 7/0 T2D 8/0	HuGene-1_1-st	'Caucasian'	-	-	Inclusion: obese men undergoing Roux-en Y gastric bypass surgery.	-	NGT 38.6±2.0* T2D 40.3±3.1*	NGT 46±7.6* T2D 41±11*	-	-

Pancreas

GEO ID, tissue & date submitted	Ca/Co (M/F)[a]	Array[b]	Ethnicity	Medication	Disease duration	Cause of death	Co-variates	BMI[c]	Age[c]	Fasting state	Family history
**GSE25724**[99] Pancreatic islets Nov 2010	NGT 4/3 T2D 3/3	HG-U133A	-	-	-	-	Age & BMI	NGT 24.8±2.5 T2D 26±2.2	NGT 58±17.3 T2D 71±9.2	-	-
**GSE76894**[100; 101] Pancreatic islets Dec 2017	NGT 83 T2D 19	HG-U133_Plus2	Donors obtained in Pisa.	-	-	-	Age & BMI	NGT 26.5±3.6 T2D 25.8±4.2	NGT 60±16.2 T2D 72±7.5	-	-
**GSE41762**[102] Pancreatic islets Oct 2012	NGT 33/24 T2D 11/9	Hu Gene-1_0-st	Nordic Islet Trans-plantation Programme	-	-	-	Age & BMI	NGT 25.4±3.1 T2D 28.5±4.6	NGT 56±10.6 T2D 59±9.3	-	-

[a]NGT: normal glucose tolerance, IR: insulin resistant, T2D: Type 2 diabetes, FH: family history (of T2D). M=Male, F=Female.

[b]Array. HG-U133_Plus2= Affymetrix Human Genome U133 Plus 2.0 Array (n=21276 genes); HG-U133A Affymetrix Human Genome U133 Array (n=13931 genes); Hu6800=Affymetrix Human Full Length HuGeneFL Array (n=6021 genes); HG_U95A=Affymetrix Human Genome U95A Array (n=9666 genes); HuGene-1_0-st=Affymetrix Human Gene 1.0 ST Array (n=20212 genes); HG_U95Av2=Affymetrix Human Genome U95 Version 2 Array (n=9667 genes); U133_X3P, Affymetrix Human X3P Array (n=21157 genes); HuGene-1_1-st=Affymetrix Human Gene 1.1 ST Array (n=20212 genes). [c]Age and BMI, mean ± standard deviation. *individual sample level statistics not available.

Supplementary Figure S2. GEO dataset correlations. Gene expression datasets showing the global correlation of gene Z scores (absolute Z>1) for all datasets. Correlation ranges from -1 (red), 0 (white) to +1 (green).

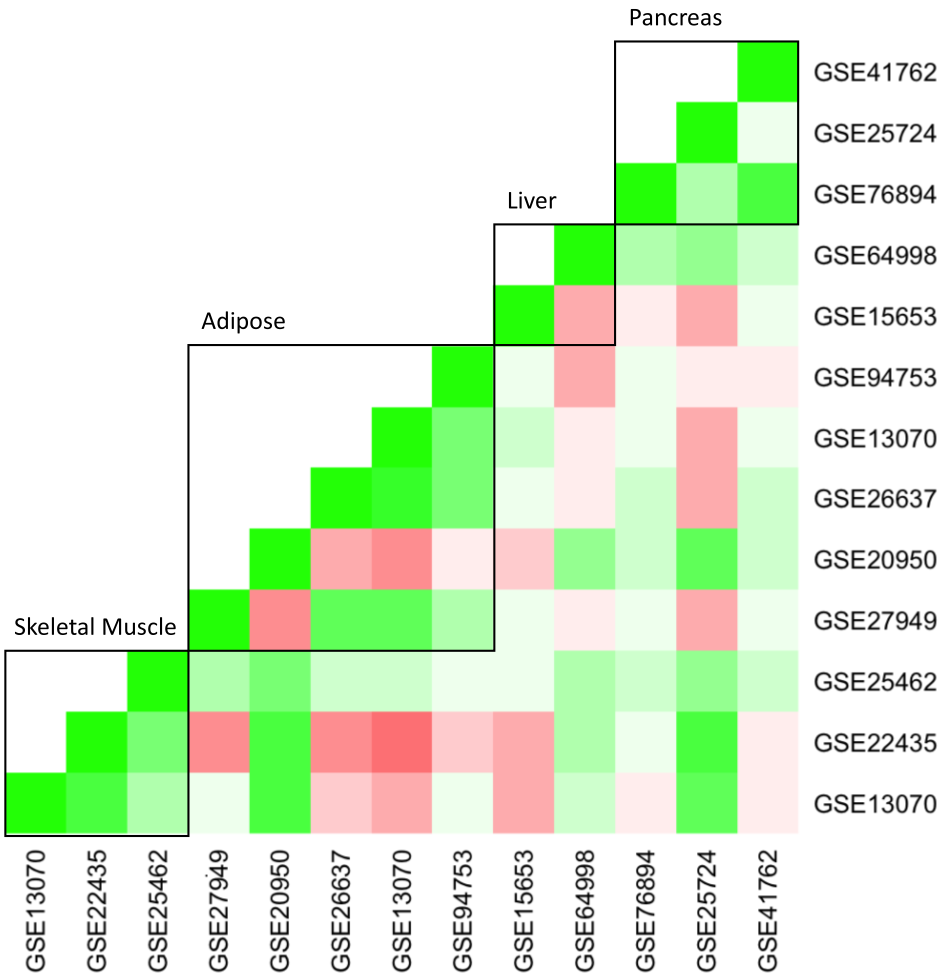

Gene set enrichment analysis

Gene set enrichment analysis was carried out using the Bioconductor R package *piano*[103]. Gene sets were loaded as ensembl gene IDs and analysis was carried out using the Wilcoxon rank-sum method with 10,000 permutations. Gene sets included the total T2D *cis*-genes (n = 763), T2D *cis*-NEMGs (n = 50) and three negative controls sets of 50 'non-T2D' NEMGs (NEMGs *not* identified as T2D *cis*-genes in this study). Gene sets representing mitochondrial pathways were downloaded from the Broad Institutes' Molecular Signatures Database (MSigDB)[104; 105], C2 Curated Gene Sets, and HGNC gene identifiers were converted to ensembl in R using *biomaRt* v.2.40.3[106].

Supplementary Figure S3. Five T2D *cis*-NEMGs on the branched chain amino acid catabolism (BCAA) pathway. The figure below is the KEGG pathway for BCAA catabolism[1]. The highlighted enzymes in yellow are encoded by five *cis*-NEMGs: *PCCA*, *MCCC1*, *ABAT*, *ACADS* and *ALDH2*.

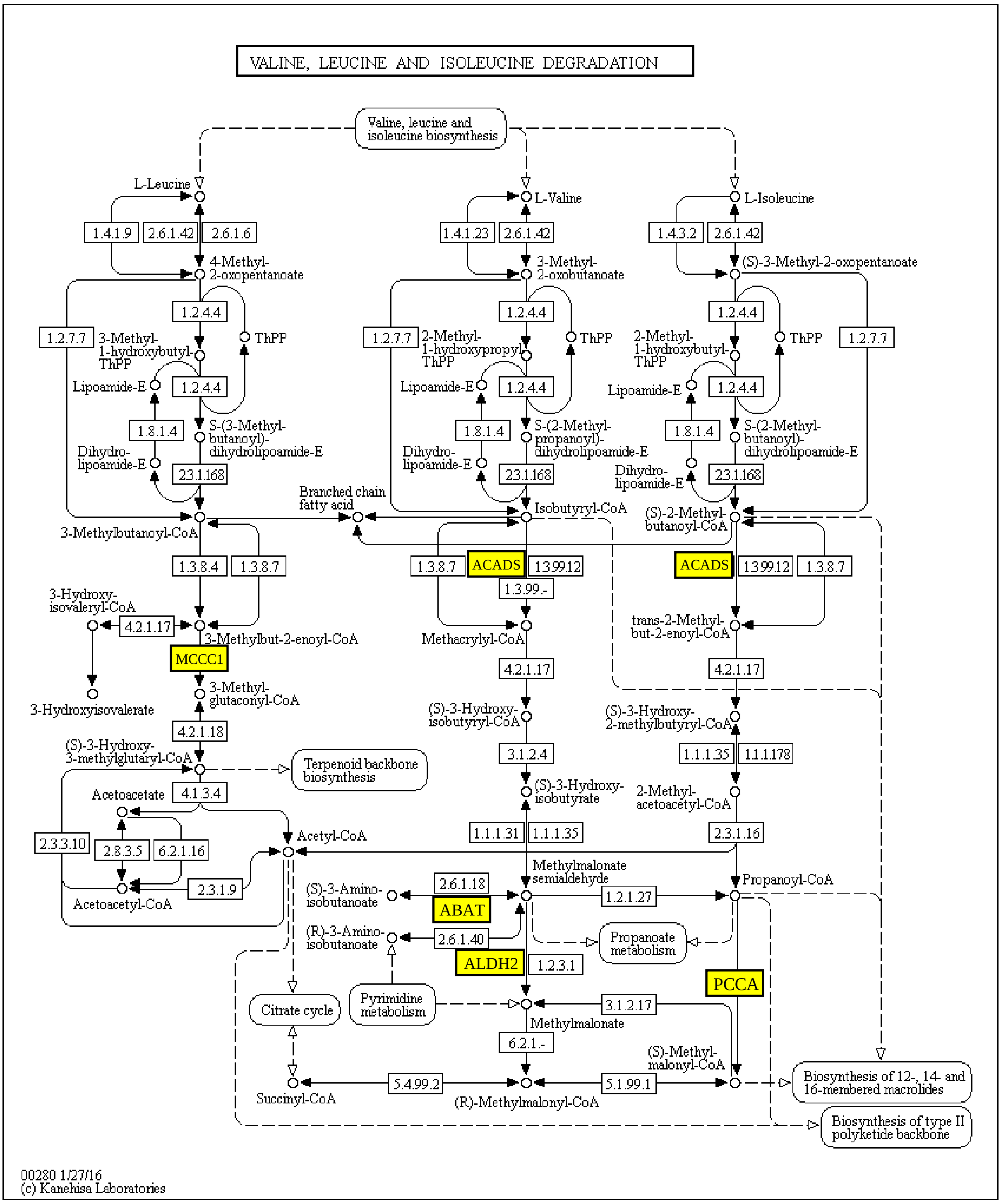

Supplementary Table S5. Gene set enrichment analysis for all GEO datasets. Gene set enrichment analysis (GSEA) results, testing differential expression of three gene sets: (1) the total T2D *cis*-genes (n = 763) and (2) the T2D *cis*-NEMGs (n = 50) against the genomic background and (3) the T2D *cis*-NEMGs against a background of all NEMGs (n = 1,204). For the first two GSEA, false discovery rate (FDR) *q*-values are presented, while uncorrected *p*-values are presented for the third analysis (this is due to the limited power to detect an enrichment of *cis*-NEMG differential expression against the background of highly correlated NEMGs). GSEA were calculated with a Wilcoxon statistic and gene sampling using 10,000 permutations. *n.s.* = not significant. ↓ indicates a significant test (*q*-value <0.05 or *p*-value <0.05) for decreased expression relative to the background, ↑ indicates significant increased expression and no arrow indicates a significant result for a test of non-directional enrichment (both increased and decreased).

Adipose (subcutaneous)

		**GSE27949**	**GSE20950**	**GSE26637**	**GSE13070**	**GSE101492**
Vs. genomic background	Total *cis*-genes (*q*-value)	↑ 0.017	↓ 2.5*e*-04	↑ 0.027	↑ 0.003	↓ 0.025
	Cis-NEMGs (*q*-value)	*n.s.*	↓<1.7*e*-04	*n.s.*	↓ 0.047	↓ 0.049
Vs. all NEMGs	T2D *cis*-NEMGs (*p*-value)	↑0.023	*n.s.*	*n.s.*	*n.s.*	*n.s.*

Skeletal muscle

		**GSE25462**	**GSE13070**	**GSE22435**
Vs. genomic background	Total *cis*-genes (*q*-value)	*n.s.*	↓ 0.054	↓ 0.002
	T2D *cis*-NEMGs (*q*-value)	↓<4.0*e*-04	↓<1.0*e*-04	↓<2.0*e*-04
Vs. all NEMGs	T2D *cis*-NEMGs (*p*-value)	↓ 0.040	*n.s.*	↓0.086

Liver

		**GSE15653**	**GSE64998**
Vs. genomic background	Total *cis*-genes (*q*-value)	*n.s.*	↓ 0.010
	Cis-NEMGs (*q*-value)	*n.s*	↓ 0.008
Vs. all NEMGs	T2D *cis*-NEMGs (*p*-value)	*n.s.*	*n.s.*

Pancreas

		**GSE76894**	**GSE25724**	**GSE41762**
Vs. genomic background	Total *cis*-genes (*q*-value)	0.035	*n.s.*	0.018
	Cis-NEMGs (*q*-value)	*n.s.*	↓<1.3*e*-04	*n.s.*
Vs. all NEMGs	T2D *cis*-NEMGs (*p*-value)	*n.s.*	↓ 0.016	↓ 0.008

Supplementary Table S6. All mitochondrial pathways tested for enrichment. A total of 41 gene sets, containing >25% of genes within the combined MitoCarta2.0[107] and MitoCarta+[108] databases, were downloaded from the Molecular Signatures Database (MSigSB) curated gene sets (C2)[105; 109]. These gene sets were subsequently tested for evidence of enrichment by count in the total 763 T2D *cis*-genes, compared to the genomic background.

**MSigDB C2 Gene Set (Source)**		
**(KEGG)**	Tryptophan metabolism	Mitochondrial tRNA aminoacylation
Valine leucine and isoleucine degradation	**(Reactome)**	Mitochondrial calcium ion transport
Butanoate metabolism	Release of apoptotic factors from the mitochondria	Pyruvate metabolism and citric acid TCA cycle
Propanoate metabolism	Purine catabolism	Mitochondrial protein import
PPAR signaling pathway	Activated AMPK stimulates FAO in muscle	Metabolism of amino acids and derivatives
Lysine degradation	Gluconeogenesis	Pyrimidine metabolism
Glycolysis and gluconeogenesis	Mitochondrial fatty acid β-oxidation	Fatty acid triacylglycerol and ketone body metabolism
Oxidative phosphorylation	Pyrimidine catabolism	Mitochondrial biogenesis
Fatty acid metabolism	Synthesis of very long chain fatty acyl CoAs	**(Others)**
Glycine, serine and threonine metabolism	Fatty-acyl CoA biosynthesis	(Mootha) mitochondria
Alanine aspartate and glutamate metabolism	TCA cycle and respiratory electron transport	(Biocarta) mitochondria pathway
Glyoxylate and dicarboxylate metabolism	Branched chain amino acid catabolism	(Wong) mitochondria gene module
Citrate / TCA cycle	Tyrosine metabolism	Biotin carboxylases (manually defined)
Beta alanine metabolism	Respiratory electron transport	
Pyruvate metabolism	Synthesis and interconversion of nucleotide di- and triphosphates	
Arginine and proline metabolism		

References

1. Kanehisa, M., Sato, Y., Kawashima, M., Furumichi, M., and Tanabe, M. (2016). KEGG as a reference resource for gene and protein annotation. Nucleic Acids Res 44, D457-462.
2. Rebhan, M., Chalifa-Caspi, V., Prilusky, J., and Lancet, D. (1997). GeneCards: integrating information about genes, proteins and diseases. Trends Genet 13, 163.
3. Geisler, C.E., Ghimire, S., Bruggink, S.M., Miller, K.E., Weninger, S.N., Kronenfeld, J.M., Yoshino, J., Klein, S., Duca, F.A., and Renquist, B.J. (2020). A Critical Role of Hepatic GABA in The Metabolic Dysfunction and Hyperphagia of Obesity. bioRxiv.
4. Ishikawa, A., Ishiguro, S., and Tamai, M. (1996). Changes in GABA metabolism in streptozotocin-induced diabetic rat retinas. Curr Eye Res 15, 63-71.
5. Soltani, N., Qiu, H., Aleksic, M., Glinka, Y., Zhao, F., Liu, R., Li, Y., Zhang, N., Chakrabarti, R., Ng, T., et al. (2011). GABA exerts protective and regenerative effects on islet beta cells and reverses diabetes. Proc Natl Acad Sci U S A 108, 11692-11697.
6. Tian, M., Mei, D., Freri, E., Hernandez, C.C., Granata, T., Shen, W., Macdonald, R.L., and Guerrini, R. (2013). Impaired surface alphabetagamma GABA(A) receptor expression in familial epilepsy due to a GABRG2 frameshift mutation. Neurobiol Dis 50, 135-141.
7. Prud'homme, G.J., Glinka, Y., Udovyk, O., Hasilo, C., Paraskevas, S., and Wang, Q. (2014). GABA protects pancreatic beta cells against apoptosis by increasing SIRT1 expression and activity. Biochem Biophys Res Commun 452, 649-654.
8. Purwana, I., Zheng, J., Li, X., Deurloo, M., Son, D.O., Zhang, Z., Liang, C., Shen, E., Tadkase, A., Feng, Z.P., et al. (2014). GABA promotes human beta-cell proliferation and modulates glucose homeostasis. Diabetes 63, 4197-4205.
9. Braun, M., Wendt, A., Birnir, B., Broman, J., Eliasson, L., Galvanovskis, J., Gromada, J., Mulder, H., and Rorsman, P. (2004). Regulated exocytosis of GABA-containing synaptic-like microvesicles in pancreatic beta-cells. J Gen Physiol 123, 191-204.
10. Dong, H., Kumar, M., Zhang, Y., Gyulkhandanyan, A., Xiang, Y.Y., Ye, B., Perrella, J., Hyder, A., Zhang, N., Wheeler, M., et al. (2006). Gamma-aminobutyric acid up- and downregulates insulin secretion from beta cells in concert with changes in glucose concentration. Diabetologia 49, 697-705.
11. Rorsman, P., Berggren, P.O., Bokvist, K., Ericson, H., Mohler, H., Ostenson, C.G., and Smith, P.A. (1989). Glucose-inhibition of glucagon secretion involves activation of GABAA-receptor chloride channels. Nature 341, 233-236.
12. Replication, D.I.G., Meta-analysis, C., Asian Genetic Epidemiology Network Type 2 Diabetes, C., South Asian Type 2 Diabetes, C., Mexican American Type 2 Diabetes, C., Type 2 Diabetes Genetic Exploration by Nex-generation sequencing in muylti-Ethnic Samples, C., Mahajan, A., Go, M.J., Zhang, W., Below, J.E., et al. (2014). Genome-wide trans-ancestry meta-analysis provides insight into the genetic architecture of type 2 diabetes susceptibility. Nat Genet 46, 234-244.
13. Harder, M.N., Appel, E.V., Grarup, N., Gjesing, A.P., Ahluwalia, T.S., Jorgensen, T., Christensen, C., Brandslund, I., Linneberg, A., Sorensen, T.I., et al. (2015). The type 2 diabetes risk allele of TMEM154-rs6813195 associates with decreased beta cell function in a study of 6,486 Danes. PLoS One 10, e0120890.
14. Greenwald, W.W., Chiou, J., Yan, J., Qiu, Y., Dai, N., Wang, A., Nariai, N., Aylward, A., Han, J.Y., Kadakia, N., et al. (2019). Pancreatic islet chromatin accessibility and conformation reveals distal enhancer networks of type 2 diabetes risk. Nat Commun 10, 2078.

15. Bian, L., Hanson, R.L., Muller, Y.L., Ma, L., Investigators, M., Kobes, S., Knowler, W.C., Bogardus, C., and Baier, L.J. (2010). Variants in ACAD10 are associated with type 2 diabetes, insulin resistance and lipid oxidation in Pima Indians. Diabetologia 53, 1349-1353.
16. Chen, D., Feng, X., Lv, Z., Xu, X., Lu, Y., Wu, W., Wu, H., Liu, H., Cao, L., Ye, S., et al. (2019). ACADS acts as a potential methylation biomarker associated with the proliferation and metastasis of hepatocellular carcinomas. Aging (Albany NY) 11, 8825-8844.
17. Ghosh, S., Kruger, C., Wicks, S., Simon, J., Kumar, K.G., Johnson, W.D., Mynatt, R.L., Noland, R.C., and Richards, B.K. (2016). Short chain acyl-CoA dehydrogenase deficiency and short-term high-fat diet perturb mitochondrial energy metabolism and transcriptional control of lipid-handling in liver. Nutr Metab (Lond) 13, 17.
18. Hornbak, M., Banasik, K., Justesen, J.M., Krarup, N.T., Sandholt, C.H., Andersson, A., Sandbaek, A., Lauritzen, T., Pisinger, C., Witte, D.R., et al. (2011). The minor C-allele of rs2014355 in ACADS is associated with reduced insulin release following an oral glucose load. BMC Med Genet 12, 4.
19. Pan, G., Deshpande, M., Thandavarayan, R.A., and Palaniyandi, S.S. (2016). ALDH2 Inhibition Potentiates High Glucose Stress-Induced Injury in Cultured Cardiomyocytes. J Diabetes Res 2016, 1390861.
20. Xu, F., Chen, Y., Lv, R., Zhang, H., Tian, H., Bian, Y., Feng, J., Sun, Y., Li, R., Wang, R., et al. (2010). ALDH2 genetic polymorphism and the risk of type II diabetes mellitus in CAD patients. Hypertens Res 33, 49-55.
21. Li, G.Y., Li, Z.B., Li, F., Dong, L.P., Tang, L., Xiang, J., Li, J.M., and Bao, M.H. (2017). Meta-Analysis on the Association of ALDH2 Polymorphisms and Type 2 Diabetic Mellitus, Diabetic Retinopathy. Int J Environ Res Public Health 14.
22. Yin, G., Naito, M., Wakai, K., Morita, E., Kawai, S., Hamajima, N., Suzuki, S., Kita, Y., Takezaki, T., Tanaka, K., et al. (2016). ALDH2 polymorphism is associated with fasting blood glucose through alcohol consumption in Japanese men. Nagoya J Med Sci 78, 183-193.
23. Zhang, Y., Babcock, S.A., Hu, N., Maris, J.R., Wang, H., and Ren, J. (2012). Mitochondrial aldehyde dehydrogenase (ALDH2) protects against streptozotocin-induced diabetic cardiomyopathy: role of GSK3beta and mitochondrial function. BMC Med 10, 40.
24. Rouzier, C., Moore, D., Delorme, C., Lacas-Gervais, S., Ait-El-Mkadem, S., Fragaki, K., Burté, F., Serre, V., Bannwarth, S., and Chaussenot, A. (2017). A novel CISD2 mutation associated with a classical Wolfram syndrome phenotype alters Ca2+ homeostasis and ER-mitochondria interactions. Human molecular genetics 26, 1599-1611.
25. Cattaneo, M., La Sala, L., Rondinelli, M., Errichiello, E., Zuffardi, O., Puca, A.A., Genovese, S., and Ceriello, A. (2017). A donor splice site mutation in CISD2 generates multiple truncated, non-functional isoforms in Wolfram syndrome type 2 patients. BMC Medical Genetics 18, 147.
26. Zhang, Y., Feng, L., Kong, X., Wu, J., Chen, Y., and Tian, G. (2019). Novel mutations and the ophthalmologic characters in Chinese patients with Wolfram Syndrome. Orphanet journal of rare diseases 14, 190.
27. Mozzillo, E., Delvecchio, M., Carella, M., Grandone, E., Palumbo, P., Salina, A., Aloi, C., Buono, P., Izzo, A., D'Annunzio, G., et al. (2014). A novel CISD2 intragenic deletion, optic neuropathy and platelet aggregation defect in Wolfram syndrome type 2. BMC Med Genet 15, 88.

28. Patel, D., Ythier, D., Brozzi, F., Eizirik, D.L., and Thorens, B. (2015). Clic4, a novel protein that sensitizes beta-cells to apoptosis. Mol Metab 4, 253-264.
29. Modi, K., Santani, D.D., Goyal, R.K., and Bhatt, P.A. (2006). Effect of coenzyme Q10 on catalase activity and other antioxidant parameters in streptozotocin-induced diabetic rats. Biol Trace Elem Res 109, 25-34.
30. Amin, M.M., Asaad, G.F., Abdel Salam, R.M., El-Abhar, H.S., and Arbid, M.S. (2014). Novel CoQ10 antidiabetic mechanisms underlie its positive effect: modulation of insulin and adiponectine receptors, Tyrosine kinase, PI3K, glucose transporters, sRAGE and visfatin in insulin resistant/diabetic rats. PLoS One 9, e89169.
31. Sourris, K.C., Harcourt, B.E., Tang, P.H., Morley, A.L., Huynh, K., Penfold, S.A., Coughlan, M.T., Cooper, M.E., Nguyen, T.V., Ritchie, R.H., et al. (2012). Ubiquinone (coenzyme Q10) prevents renal mitochondrial dysfunction in an experimental model of type 2 diabetes. Free Radic Biol Med 52, 716-723.
32. Sun, I.O., Jin, L., Jin, J., Lim, S.W., Chung, B.H., and Yang, C.W. (2019). The effects of addition of coenzyme Q10 to metformin on sirolimus-induced diabetes mellitus. Korean J Intern Med 34, 365-374.
33. Zahedi, H., Eghtesadi, S., Seifirad, S., Rezaee, N., Shidfar, F., Heydari, I., Golestan, B., and Jazayeri, S. (2014). Effects of CoQ10 Supplementation on Lipid Profiles and Glycemic Control in Patients with Type 2 Diabetes: a randomized, double blind, placebo-controlled trial. J Diabetes Metab Disord 13, 81.
34. Merino, J., Leong, A., Liu, C.-T., Porneala, B., Walford, G.A., von Grotthuss, M., Wang, T.J., Flannick, J., Dupuis, J., and Levy, D. (2018). Metabolomics insights into early type 2 diabetes pathogenesis and detection in individuals with normal fasting glucose. Diabetologia 61, 1315-1324.
35. Locke, A.E., Kahali, B., Berndt, S.I., Justice, A.E., Pers, T.H., Day, F.R., Powell, C., Vedantam, S., Buchkovich, M.L., Yang, J., et al. (2015). Genetic studies of body mass index yield new insights for obesity biology. Nature 518, 197-206.
36. Mahajan, A., Taliun, D., Thurner, M., Robertson, N.R., Torres, J.M., Rayner, N.W., Payne, A.J., Steinthorsdottir, V., Scott, R.A., and Grarup, N. (2018). Fine-mapping type 2 diabetes loci to single-variant resolution using high-density imputation and islet-specific epigenome maps. Nature genetics 50, 1505-1513.
37. Jager, S., Wahl, S., Kroger, J., Sharma, S., Hoffmann, P., Floegel, A., Pischon, T., Prehn, C., Adamski, J., Muller-Nurasyid, M., et al. (2017). Genetic variants including markers from the exome chip and metabolite traits of type 2 diabetes. Sci Rep 7, 6037.
38. Swan, E.J., Salem, R.M., Sandholm, N., Tarnow, L., Rossing, P., Lajer, M., Groop, P.H., Maxwell, A.P., McKnight, A.J., and Consortium, G. (2015). Genetic risk factors affecting mitochondrial function are associated with kidney disease in people with Type 1 diabetes. Diabet Med 32, 1104-1109.
39. Miller, R.A., Shi, Y., Lu, W., Pirman, D.A., Jatkar, A., Blatnik, M., Wu, H., Cardenas, C., Wan, M., Foskett, J.K., et al. (2018). Targeting hepatic glutaminase activity to ameliorate hyperglycemia. Nat Med 24, 518-524.
40. Scott, R.A., Scott, L.J., Magi, R., Marullo, L., Gaulton, K.J., Kaakinen, M., Pervjakova, N., Pers, T.H., Johnson, A.D., Eicher, J.D., et al. (2017). An Expanded Genome-Wide Association Study of Type 2 Diabetes in Europeans. Diabetes 66, 2888-2902.
41. Suhre, K., Shin, S.Y., Petersen, A.K., Mohney, R.P., Meredith, D., Wagele, B., Altmaier, E., CardioGram, Deloukas, P., Erdmann, J., et al. (2011). Human metabolic individuality in biomedical and pharmaceutical research. Nature 477, 54-60.
42. Hammond, L.E., Neschen, S., Romanelli, A.J., Cline, G.W., Ilkayeva, O.R., Shulman, G.I., Muoio, D.M., and Coleman, R.A. (2005). Mitochondrial glycerol-3-phosphate

acyltransferase-1 is essential in liver for the metabolism of excess acyl-CoAs. Journal of Biological Chemistry 280, 25629-25636.
43. Nagle, C.A., An, J., Shiota, M., Torres, T.P., Cline, G.W., Liu, Z.X., Wang, S., Catlin, R.L., Shulman, G.I., Newgard, C.B., et al. (2007). Hepatic overexpression of glycerol-sn-3-phosphate acyltransferase 1 in rats causes insulin resistance. J Biol Chem 282, 14807-14815.
44. Zhang, C., Wendel, A.A., Keogh, M.R., Harris, T.E., Chen, J., and Coleman, R.A. (2012). Glycerolipid signals alter mTOR complex 2 (mTORC2) to diminish insulin signaling. Proc Natl Acad Sci U S A 109, 1667-1672.
45. Zhang, C., Cooper, D.E., Grevengoed, T.J., Li, L.O., Klett, E.L., Eaton, J.M., Harris, T.E., and Coleman, R.A. (2014). Glycerol-3-phosphate acyltransferase-4-deficient mice are protected from diet-induced insulin resistance by the enhanced association of mTOR and rictor. Am J Physiol Endocrinol Metab 307, E305-315.
46. Xu, H., Wilcox, D., Nguyen, P., Voorbach, M., Suhar, T., Morgan, S.J., An, W.F., Ge, L., Green, J., Wu, Z., et al. (2006). Hepatic knockdown of mitochondrial GPAT1 in ob/ob mice improves metabolic profile. Biochem Biophys Res Commun 349, 439-448.
47. Yuan, J., Dunn, P., and Martinus, R.D. (2011). Detection of Hsp60 in saliva and serum from type 2 diabetic and non-diabetic control subjects. Cell Stress and Chaperones 16, 689.
48. Oksala, N.K., Laaksonen, D.E., Lappalainen, J., Khanna, S., Nakao, C., Hänninen, O., Sen, C.K., and Atalay, M. (2006). Heat shock protein 60 response to exercise in diabetes: Effects of α-lipoic acid supplementation. Journal of Diabetes and its Complications 20, 257-261.
49. Martinus, R.D., and Goldsbury, J. (2018). Endothelial TNF-α induction by Hsp60 secreted from THP-1 monocytes exposed to hyperglycaemic conditions. Cell Stress and Chaperones 23, 519-525.
50. Aluksanasuwan, S., Sueksakit, K., Fong-Ngern, K., and Thongboonkerd, V. (2017). Role of HSP60 (HSPD1) in diabetes-induced renal tubular dysfunction: regulation of intracellular protein aggregation, ATP production, and oxidative stress. FASEB J 31, 2157-2167.
51. Kleinridders, A., Lauritzen, H.P., Ussar, S., Christensen, J.H., Mori, M.A., Bross, P., and Kahn, C.R. (2013). Leptin regulation of Hsp60 impacts hypothalamic insulin signaling. J Clin Invest 123, 4667-4680.
52. Raz, I., Elias, D., Avron, A., Tamir, M., Metzger, M., and Cohen, I.R. (2001). β-cell function in new-onset type 1 diabetes and immunomodulation with a heat-shock protein peptide (DiaPep277): a randomised, double-blind, phase II trial. The Lancet 358, 1749-1753.
53. Sarikonda, G., Sachithanantham, S., Miller, J.F., Pagni, P.P., Coppieters, K.T., and von Herrath, M. (2015). The Hsp60 peptide p277 enhances anti-CD3 mediated diabetes remission in non-obese diabetic mice. Journal of Autoimmunity 59, 61-66.
54. Verrijn Stuart, A., de Jager, W., Klein, M., Teklenburg, G., Nuboer, R., Hoorweg, J., de Vroede, M., de Kruijff, I., Fick, M., and Schroor, E. (2012). Recognition of heat shock protein 60 epitopes in children with type 1 diabetes. Diabetes/metabolism research and reviews 28, 527-534.
55. Huurman, V., Van Der Meide, P., Duinkerken, G., Willemen, S., Cohen, I., Elias, D., and Roep, B. (2008). Immunological efficacy of heat shock protein 60 peptide DiaPep277TM therapy in clinical type I diabetes. Clinical & Experimental Immunology 152, 488-497.

56. Elias, D., Marcus, H., Reshef, T., Ablamunits, V., and Cohen, I.R. (1995). Induction of diabetes in standard mice by immunization with the p277 peptide of a 60-kDa heat shock protein. European journal of immunology 25, 2851-2857.
57. Zhang, D., Liu, H., Zhang, Y., Li, J., Fu, Y., Zheng, Y., Wu, J., Ma, M., Wen, Z., and Wang, C. (2020). Heat shock protein 60 (HSP60) modulates adiponectin signaling by stabilizing adiponectin receptor. Cell Communication and Signaling 18, 1-9.
58. Chae, S., Kim, S.-J., Do Koo, Y., Lee, J.H., Kim, H., Ahn, B.Y., Ha, Y.-C., Kim, Y.-H., Jang, M.G., and Koo, K.-H. (2018). A mitochondrial proteome profile indicative of type 2 diabetes mellitus in skeletal muscles. Experimental & molecular medicine 50, 1-14.
59. MacDonald, M.J., Brown, L.J., Longacre, M.J., Stoker, S.W., and Kendrick, M.A. (2013). Knockdown of both mitochondrial isocitrate dehydrogenase enzymes in pancreatic beta cells inhibits insulin secretion. Biochimica et Biophysica Acta (BBA)-General Subjects 1830, 5104-5111.
60. Timmons, J.A., Atherton, P.J., Larsson, O., Sood, S., Blokhin, I.O., Brogan, R.J., Volmar, C.-H., Josse, A.R., Slentz, C., and Wahlestedt, C. (2018). A coding and non-coding transcriptomic perspective on the genomics of human metabolic disease. Nucleic acids research 46, 7772-7792.
61. Pietiläinen, K.H., Naukkarinen, J., Rissanen, A., Saharinen, J., Ellonen, P., Keränen, H., Suomalainen, A., Götz, A., Suortti, T., and Yki-Järvinen, H. (2008). Global transcript profiles of fat in monozygotic twins discordant for BMI: pathways behind acquired obesity. PLoS Med 5, e51.
62. Wiklund, P., Zhang, X., Pekkala, S., Autio, R., Kong, L., Yang, Y., Keinänen-Kiukaanniemi, S., Alen, M., and Cheng, S. (2016). Insulin resistance is associated with altered amino acid metabolism and adipose tissue dysfunction in normoglycemic women. Scientific reports 6, 1-11.
63. Anderson, K.A., Huynh, F.K., Fisher-Wellman, K., Stuart, J.D., Peterson, B.S., Douros, J.D., Wagner, G.R., Thompson, J.W., Madsen, A.S., and Green, M.F. (2017). SIRT4 is a lysine deacylase that controls leucine metabolism and insulin secretion. Cell metabolism 25, 838-855. e815.
64. Sheng, J., Li, H., Dai, Q., Lu, C., Xu, M., Zhang, J., and Feng, J. (2018). NR4A1 promotes diabetic nephropathy by activating mff-mediated mitochondrial fission and suppressing parkin-mediated mitophagy. Cellular Physiology and Biochemistry 48, 1675-1693.
65. Lee, J., Pappalardo, Z., Chopra, D.G., Hennings, T.G., Vaughn, I., Lan, C., Choe, J.J., Ang, K., Chen, S., and Arkin, M. (2018). A genetic interaction map of insulin production identifies Mfi as an inhibitor of mitochondrial fission. Endocrinology 159, 3321-3330.
66. Bosma, M., Hesselink, M.K., Sparks, L.M., Timmers, S., Ferraz, M.J., Mattijssen, F., van Beurden, D., Schaart, G., de Baets, M.H., and Verheyen, F.K. (2012). Perilipin 2 improves insulin sensitivity in skeletal muscle despite elevated intramuscular lipid levels. Diabetes 61, 2679-2690.
67. Quilter, C.R., Cooper, W.N., Cliffe, K.M., Skinner, B.M., Prentice, P.M., Nelson, L., Bauer, J., Ong, K.K., Constância, M., and Lowe, W.L. (2014). Impact on offspring methylation patterns of maternal gestational diabetes mellitus and intrauterine growth restraint suggest common genes and pathways linked to subsequent type 2 diabetes risk. The FASEB Journal 28, 4868-4879.
68. Wu, C., Xu, G., Tsai, S.-Y.A., Freed, W.J., and Lee, C.-T. (2017). Transcriptional profiles of type 2 diabetes in human skeletal muscle reveal insulin resistance, metabolic defects, apoptosis, and molecular signatures of immune activation in

response to infections. Biochemical and biophysical research communications 482, 282-288.
69. Dahlman, I., Forsgren, M., Sjögren, A., Nordström, E.A., Kaaman, M., Näslund, E., Attersand, A., and Arner, P. (2006). Downregulation of electron transport chain genes in visceral adipose tissue in type 2 diabetes independent of obesity and possibly involving tumor necrosis factor-α. Diabetes 55, 1792-1799.
70. Wen, L., Zhang, Z., Peng, R., Zhang, L., Liu, H., Peng, H., and Sun, Y. (2019). Whole transcriptome analysis of diabetic nephropathy in the db/db mouse model of type 2 diabetes. Journal of cellular biochemistry 120, 17520-17533.
71. Ling, C., Poulsen, P., Simonsson, S., Rönn, T., Holmkvist, J., Almgren, P., Hagert, P., Nilsson, E., Mabey, A.G., and Nilsson, P. (2007). Genetic and epigenetic factors are associated with expression of respiratory chain component NDUFB6 in human skeletal muscle. The Journal of clinical investigation 117, 3427-3435.
72. Sreekumar, R., Halvatsiotis, P., Schimke, J.C., and Nair, K.S. (2002). Gene expression profile in skeletal muscle of type 2 diabetes and the effect of insulin treatment. Diabetes 51, 1913-1920.
73. Snogdal, L.S., Wod, M., Grarup, N., Vestmar, M., Sparsø, T., Jørgensen, T., Lauritzen, T., Beck-Nielsen, H., Henriksen, J., and Pedersen, O. (2012). Common variation in oxidative phosphorylation genes is not a major cause of insulin resistance or type 2 diabetes. Diabetologia 55, 340-348.
74. Merla, G., Howald, C., Antonarakis, S.E., and Reymond, A. (2004). The subcellular localization of the ChoRE-binding protein, encoded by the Williams–Beuren syndrome critical region gene 14, is regulated by 14-3-3. Human molecular genetics 13, 1505-1514.
75. Wang, X., Lai, S., Ye, Y., Hu, Y., Pan, D., Bai, X., and Shen, J. (2020). Conditional knockout of pyruvate dehydrogenase in mouse pancreatic β-cells causes morphological and functional changes. Molecular Medicine Reports 21, 1717-1726.
76. Liu, X., Xu, C., Xu, L., Li, X., Sun, H., Xue, M., Li, T., Yu, X., Sun, B., and Chen, L. (2020). Empagliflozin improves diabetic renal tubular injury by alleviating mitochondrial fission via AMPK/SP1/PGAM5 pathway. Metabolism 111, 154334.
77. Sekine, S., Yao, A., Hattori, K., Sugawara, S., Naguro, I., Koike, M., Uchiyama, Y., Takeda, K., and Ichijo, H. (2016). The ablation of mitochondrial protein phosphatase Pgam5 confers resistance against metabolic stress. EBioMedicine 5, 82-92.
78. Kraja, A.T., Liu, C., Fetterman, J.L., Graff, M., Have, C.T., Gu, C., Yanek, L.R., Feitosa, M.F., Arking, D.E., and Chasman, D.I. (2019). Associations of mitochondrial and nuclear mitochondrial variants and genes with seven metabolic traits. The American Journal of Human Genetics 104, 112-138.
79. Yoshikumi, Y., Mashima, H., Ueda, N., Ohno, H., Suzuki, J., Tanaka, S., Hayashi, M., Sekine, N., Ohnishi, H., and Yasuda, H. (2005). Roles of CTPL/Sfxn3 and Sfxn family members in pancreatic islet. Journal of cellular biochemistry 95, 1157-1168.
80. Uyy, E., Suica, V.I., Boteanu, R.M., Safciuc, F., Cerveanu-Hogas, A., Ivan, L., Stavaru, C., Simionescu, M., and Antohe, F. (2020). Diabetic nephropathy associates with deregulation of enzymes involved in kidney sulphur metabolism. Journal of cellular and molecular medicine.
81. Hakonarson, H., Qu, H.-Q., Bradfield, J.P., Marchand, L., Kim, C.E., Glessner, J.T., Grabs, R., Casalunovo, T., Taback, S.P., and Frackelton, E.C. (2008). A novel susceptibility locus for type 1 diabetes on Chr12q13 identified by a genome-wide association study. Diabetes 57, 1143-1146.

82. Plagnol, V., Howson, J.M., Smyth, D.J., Walker, N., Hafler, J.P., Wallace, C., Stevens, H., Jackson, L., Simmonds, M.J., and Bingley, P.J. (2011). Genome-wide association analysis of autoantibody positivity in type 1 diabetes cases. PLoS Genet 7, e1002216.
83. Wallace, C., Rotival, M., Cooper, J.D., Rice, C.M., Yang, J.H., McNeill, M., Smyth, D.J., Niblett, D., Cambien, F., and Consortium, C. (2012). Statistical colocalization of monocyte gene expression and genetic risk variants for type 1 diabetes. Human molecular genetics 21, 2815-2824.
84. Carlson, M., Falcon, S., Pages, H., and Li, N. (2016). hgu133plus2. db: Affymetrix Human Genome U133 Plus 2.0 Array annotation data (chip hgu133plus2). R package version 3.
85. Carlson, M., Falcon, S., Pages, H., and Li, N. (2013). hu6800. db: Affymetrix HuGeneFL Genome Array annotation data (chip hu6800). R package version 2.
86. JW, M. (2017). hugene10stprobeset.db: Affymetrix hugene10 annotation data (chip hugene10stprobeset). R package version 8.7.0.
87. B, C. (2015). pd.hg.u95av2: Platform Design Info for The Manufacturer's Name HG_U95Av2. . R package version 3.12.0.
88. Carvalho, B.S., and Irizarry, R.A. (2010). A framework for oligonucleotide microarray preprocessing. Bioinformatics 26, 2363-2367.
89. Choi, J.K., Yu, U., Kim, S., and Yoo, O.J. (2003). Combining multiple microarray studies and modeling interstudy variation. Bioinformatics 19 Suppl 1, i84-90.
90. Sears, D.D., Hsiao, G., Hsiao, A., Yu, J.G., Courtney, C.H., Ofrecio, J.M., Chapman, J., and Subramaniam, S. (2009). Mechanisms of human insulin resistance and thiazolidinedione-mediated insulin sensitization. Proc Natl Acad Sci U S A 106, 18745-18750.
91. Pihlajamäki, J., Lerin, C., Itkonen, P., Boes, T., Floss, T., Schroeder, J., Dearie, F., Crunkhorn, S., Burak, F., and Jimenez-Chillaron, J.C. (2011). Expression of the splicing factor gene SFRS10 is reduced in human obesity and contributes to enhanced lipogenesis. Cell metabolism 14, 208-218.
92. Jin, W., Goldfine, A.B., Boes, T., Henry, R.R., Ciaraldi, T.P., Kim, E.Y., Emecan, M., Fitzpatrick, C., Sen, A., Shah, A., et al. (2011). Increased SRF transcriptional activity in human and mouse skeletal muscle is a signature of insulin resistance. J Clin Invest 121, 918-929.
93. Keller, P., Gburcik, V., Petrovic, N., Gallagher, I.J., Nedergaard, J., Cannon, B., and Timmons, J.A. (2011). Gene-chip studies of adipogenesis-regulated microRNAs in mouse primary adipocytes and human obesity. BMC Endocr Disord 11, 7.
94. Hardy, O.T., Perugini, R.A., Nicoloro, S.M., Gallagher-Dorval, K., Puri, V., Straubhaar, J., and Czech, M.P. (2011). Body mass index-independent inflammation in omental adipose tissue associated with insulin resistance in morbid obesity. Surg Obes Relat Dis 7, 60-67.
95. Soronen, J., Laurila, P.P., Naukkarinen, J., Surakka, I., Ripatti, S., Jauhiainen, M., Olkkonen, V.M., and Yki-Jarvinen, H. (2012). Adipose tissue gene expression analysis reveals changes in inflammatory, mitochondrial respiratory and lipid metabolic pathways in obese insulin-resistant subjects. BMC Med Genomics 5, 9.
96. Kulyte, A., Ehrlund, A., Arner, P., and Dahlman, I. (2017). Global transcriptome profiling identifies KLF15 and SLC25A10 as modifiers of adipocytes insulin sensitivity in obese women. PLoS One 12, e0178485.
97. Pihlajamaki, J., Boes, T., Kim, E.Y., Dearie, F., Kim, B.W., Schroeder, J., Mun, E., Nasser, I., Park, P.J., Bianco, A.C., et al. (2009). Thyroid hormone-related regulation of gene expression in human fatty liver. J Clin Endocrinol Metab 94, 3521-3529.

98. Kirchner, H., Sinha, I., Gao, H., Ruby, M.A., Schonke, M., Lindvall, J.M., Barres, R., Krook, A., Naslund, E., Dahlman-Wright, K., et al. (2016). Altered DNA methylation of glycolytic and lipogenic genes in liver from obese and type 2 diabetic patients. Mol Metab 5, 171-183.
99. Dominguez, V., Raimondi, C., Somanath, S., Bugliani, M., Loder, M.K., Edling, C.E., Divecha, N., da Silva-Xavier, G., Marselli, L., Persaud, S.J., et al. (2011). Class II phosphoinositide 3-kinase regulates exocytosis of insulin granules in pancreatic beta cells. J Biol Chem 286, 4216-4225.
100. Khamis, A., Canouil, M., Siddiq, A., Crouch, H., Falchi, M., Bulow, M.V., Ehehalt, F., Marselli, L., Distler, M., Richter, D., et al. (2019). Laser capture microdissection of human pancreatic islets reveals novel eQTLs associated with type 2 diabetes. Mol Metab 24, 98-107.
101. Solimena, M., Schulte, A.M., Marselli, L., Ehehalt, F., Richter, D., Kleeberg, M., Mziaut, H., Knoch, K.P., Parnis, J., Bugliani, M., et al. (2018). Systems biology of the IMIDIA biobank from organ donors and pancreatectomised patients defines a novel transcriptomic signature of islets from individuals with type 2 diabetes. Diabetologia 61, 641-657.
102. Mahdi, T., Hanzelmann, S., Salehi, A., Muhammed, S.J., Reinbothe, T.M., Tang, Y., Axelsson, A.S., Zhou, Y., Jing, X., Almgren, P., et al. (2012). Secreted frizzled-related protein 4 reduces insulin secretion and is overexpressed in type 2 diabetes. Cell Metab 16, 625-633.
103. Väremo, L., Nielsen, J., and Nookaew, I. (2013). Enriching the gene set analysis of genome-wide data by incorporating directionality of gene expression and combining statistical hypotheses and methods. Nucleic acids research 41, 4378-4391.
104. Liberzon, A., Subramanian, A., Pinchback, R., Thorvaldsdóttir, H., Tamayo, P., and Mesirov, J.P. (2011). Molecular signatures database (MSigDB) 3.0. Bioinformatics 27, 1739-1740.
105. Subramanian, T. (2005). Molecular Signatures Database (MSigDB). PNAS 102, 15545-15550.
106. Durinck, S., Moreau, Y., Kasprzyk, A., Davis, S., De Moor, B., Brazma, A., and Huber, W. (2005). BioMart and Bioconductor: a powerful link between biological databases and microarray data analysis. Bioinformatics 21, 3439-3440.
107. Calvo, S.E., Clauser, K.R., and Mootha, V.K. (2016). MitoCarta2.0: an updated inventory of mammalian mitochondrial proteins. Nucleic Acids Res 44, D1251-1257.
108. Floyd, B.J., Wilkerson, E.M., Veling, M.T., Minogue, C.E., Xia, C., Beebe, E.T., Wrobel, R.L., Cho, H., Kremer, L.S., Alston, C.L., et al. (2016). Mitochondrial Protein Interaction Mapping Identifies Regulators of Respiratory Chain Function. Mol Cell 63, 621-632.
109. Liberzon, A., Subramanian, A., Pinchback, R., Thorvaldsdottir, H., Tamayo, P., and Mesirov, J.P. (2011). Molecular signatures database (MSigDB) 3.0. Bioinformatics 27, 1739-1740.
